# Glycyrrhizin Attenuates *Salmonella enterica* Serovar Typhimurium Infection: New Insights Into Its Protective Mechanism

**DOI:** 10.3389/fimmu.2018.02321

**Published:** 2018-10-16

**Authors:** Xiaogang Xu, Li Gong, Baikui Wang, Yanping Wu, Yang Wang, Xiaoqiang Mei, Han Xu, Li Tang, Rongrong Liu, Zhonghua Zeng, Yulong Mao, Weifen Li

**Affiliations:** ^1^Key Laboratory of Molecular Animal Nutrition of the Ministry of Education, Institute of Feed Science, College of Animal Sciences, Zhejiang University, Hangzhou, China; ^2^Zhejiang Hospital & Zhejiang Provincial Key Lab of Geriatrics, Hangzhou, China

**Keywords:** glycyrrhizin, intestine, *Salmonella*, immunoprotection, dendritic cell

## Abstract

Glycyrrhizin (GL), a triterpenoid glycoside, serves important functions in various biological activities, including antiviral and antitumor immune responses. However, the anti-inflammatory effects of GL on *Salmonella enterica* serovar Typhimurium (ST)-induced injury in mice and the mechanisms underlying the protection of GL are poorly understood. Here, we investigated the effects of GL on host immune responses against ST infection in mice. A phenotypic analysis using hematoxylin and eosin (H&E) staining and transmission electron microscopy showed that GL relieved ST-induced weight loss and intestinal mucosal injury. A colonization assay showed that GL significantly reduced ST colonization in the ileum and colon and translocation to the liver and spleen. An antibacterial activity assay and real-time PCR revealed that GL had no direct inhibitory impact on ST growth or virulence gene expression. ELISA showed that GL pretreatment significantly decreased proinflammatory cytokine (IFN-γ, TNF-α, IL-6) secretion and increased anti-inflammatory cytokine (IL-10) secretion in the ileum, colon and serum of ST-infected mice. Moreover, flora analysis showed that GL reduced *Akkermansia, Sutterella, Prevotella* and *Coprococcus* but enriched *Parabacteroides* and *Anaerotruncus* in the cecum of ST-infected mice. These results suggest that GL promotes the secretion of immune factors and modulates intestinal flora to prevent further ST infection. We also analyzed the effect of GL on immunocytes and found that GL promoted the phenotypic and functional maturation of murine bone marrow-derived dendritic cells (BMDCs). Flow cytometry and western blotting demonstrated that NF-κB, ERK, and p38 MAPK were required for GL-induced BMDC maturation. The above findings indicate that GL attenuates ST infection by modulating immune function and intestinal flora. This study enriches our current knowledge of GL-mediated immunological function and provides a new perspective on the prevention of *Salmonella* infection in animals and humans.

## Introduction

Glycyrrhizin (GL), a triterpenoid glycoside, is the major biologically active component of licorice (1). Previous studies have reported that GL has important functions in various biological activities, such as immune regulation, apoptosis, oxidative stress, autophagy, tissue regeneration and cell proliferation (2–6). Accumulating evidence suggests that GL and its metabolite play an important role in antibacterial activities (7–9). GL can effectively relieve enterotoxigenic *Escherichia coli-*induced diarrhea by hampering the interaction between heat-labile enterotoxin and the ganglioside GM1 on the surfaces of mouse intestinal epithelial cells *in vitro* and *in vivo* (10). GL metabolite has bactericidal activity against penicillin-resistant *Staphylococcus aureus* and inhibits the expression of virulence genes (11). Moreover, deglycyrrhizinated licorice root extracts (DG-LRE) significantly inhibited biofilm formation by *S. mutants* UA159 (12).

Recent studies have found that GL is also particularly important in immunological activities. First, GL has distinct immunosuppressive functions; GL can inhibit the prostaglandin E2 produced by macrophages (13). GL has a glucocorticoid-like inhibitory effect on IL-8 production in A549 lung epithelial cells (14). Moreover, GL enhances the production of IL-10 induced by ConA in mouse liver dendritic cells (DCs) (15). Furthermore, GL pretreatment reduces the expression of the proinflammatory cytokines IL-1β and TNF-α in lipopolysaccharide (LPS)-induced acute lung injury in mice (16). In contrast, GL also has immune activation functions; GL elicits a dose-dependent increase in NO production and in the level of inducible nitric oxide synthase (iNOS) mRNA by regulating the NF-κB pathway in mouse macrophages (17). GL induces the production of interferon (IFN) and enhances IL-1 expression under the synergistic effect of macrophages and lymphocytes (18). GL selectively promotes the proliferation and activity of T helper (Th) cells, increases the proportion of CD4^+^ and CD8^+^ cells, activates extrathymic T lymphocytes and enhances Fas-mediated apoptosis (19, 20). Moreover, GL increases the production of IL-2 and IFN-γ in T lymphocytes and inhibits the production of IL-4 and IL-10 (21, 22). Furthermore, GL positively modulates murine DCs and has the capacity to upregulate the allostimulatory activity of DCs and induce Th1 responses (23, 24). The above findings indicate that GL has dual functions in immune regulation.

*Salmonella*, one of the most common foodborne pathogens, has strong pathogenicity and poses great threats to human and animal health (25). The prevention and control of *Salmonella* has been an increasingly serious health problem worldwide. Research on *Salmonella* treatment has mainly focused on antibiotics, antimicrobial peptides, immune adjuvants and vaccines (26–31). GL serves as a potent immune modulator with the ability to regulate the immune response (32). However, the effects of GL on *Salmonella* infection are obscure. In this report, therefore, we aimed to investigate the effects of GL on *Salmonella* infection in mice and explore the mechanisms underlying these effects.

## Materials and methods

### Experimental mice

Six- to eight-week-old female C57BL/6 mice were obtained from Slac Animal Inc. (Shanghai, China) and maintained in the Experimental Animal Center of Zhejiang University. All the animal experiments were performed in accordance with legal regulations and approved by the Institutional Animal Care and Use Committee of Zhejiang University.

### Reagents

GL was purchased from Sigma-Aldrich (Sigma-Aldrich, USA). Gentamycin (GM) was obtained from Amersco (Amersco, USA). LPS (*Escherichia coli* 0111:B4) and FITC-dextran (40,000 Da) were purchased from Sigma Chemical Co. (St. Louis, MO, USA). Recombinant mouse GM-CSF and IL-4 were obtained from PeproTech Inc. (RockyHill, NJ, USA). Anti-mouse antibodies FITC-CD11c, -CD40, -CD80, -CD83 and -CD86, Alexa Fluor®647-TLR2 and PE-MHC-II as well as anti-NF-κB p65 were obtained from Biolegend (San Diego, CA, USA). The ELISA kits for IFN-γ, TNF-α, NO, IL-6, IL-10 and IL-12p70 were obtained from eBioscience (San Diego, CA, USA). Antibodies against β-actin, phospho-JNK, JNK, phospho-p38, p38, IκBα, LaminB1 and HRP-conjugated anti-mouse and anti-goat IgG were purchased from SantaCruz Biotech (Santa Cruz, CA, USA), and phospho-ERK1/2 and anti-ERK1/2 antibodies were obtained from BD Pharmingen (San Jose, CA, USA). The inhibitors BAY 11-7082, SP600125, SB203580 and U0126 were purchased from Beyotime Biotechnology (Haimen, Jiangsu, China).

### Purification and preparation of ST

The *S*. Typhimurium strains were cultured and collected using standard batch culture procedures. The single colony of ST was isolated from a streaked LB agar plate and grown in fresh LB broth with shaking in a 37°C incubator for 12–16 h. The overnight cultures were diluted 1:5000 into 100 ml fresh sterile LB medium and harvested in logarithmic phase of growth by centrifugation at 5000 rpm for 10 min, and then washed three times with sterile PBS. The prepared ST cells were diluted with PBS to reach appropriate concentration for further study.

### GL protection assay

A total of 50 six- to eight-week-old female C57BL/6 mice with similar initial weights were randomly allocated to five groups with ten replicates in each group, including Control, ST, GL, GL plus ST and GM plus ST group. In the pretreatment stage, dose curve response of ST and GL were performed, ST (4 × 10^8^ CFU) and GL (80 mg/kg) were considered to be optimal concentrations for subsequent experiments (Supplemental Figure S1). In current study, all mice were fed a basal diet and weighted every 3 days. Control and ST group were fed sterile water, GL and GL plus ST group fed sterile water containing 0.4 mg/ml GL (80 mg/kg weight), and GM plus ST group was fed sterile water containing 0.4 mg/ml GM (80 mg/kg weight) every day. At day 21, the mice in ST, GL plus ST and GM plus ST groups were infected orally with 200 μl 2 × 10^9^ CFU/ml *S*. Typhimurium suspension by gavage, while the mice in Control and GL groups was administered with the same amount of sterile PBS (Supplemental Figure S2B). Body weight changes were measured on day 3 post challenge, and spleen, liver, blood, colon, cecum, ileum and mesenteric lymph node (MLN) were collected for further analysis, such as histopathology, bacterial translocation, gene expression and the inflammatory response.

### H&E staining

At necropsy, tissue samples of colon and ileum were collected and fixed in 4% paraformaldehyde, dehydrated and processed into paraffin sections according to a standard procedure. The paraffin sections were subjected with hematoxylin and eosin (H&E) staining for histopathology analysis.

### Determination of ST colonization

The colonization of ST in the ileum, colon, liver and spleen were detected as previously described (33). Briefly, samples were collected aseptically and homogenized in PBS containing 0.1% Triton X-100. Serial dilutions of organ homogenates were coated on SS agar plates. The CFU were quantified by visual counting of micro-colonies.

### Assay for antibacterial activity *in vitro*

The antibacterial activity of GL was determined by disc agar-diffusion method. Overnight incubation cultures of ST (1 × 10^8^ CFU) were spread on 150 ml LB broth agar medium. Agar wells were cut out using sterile cork borer with 10 mm diameter. 250 μl of different concentrations of GL (0, 50, 100, 200 μg/ml) and 25 μg/ml GM were added to different wells in the plate, and the plates were incubated at 37°C overnight. The diameter of inhibition zones was recorded in mm. Each experiment was carried out in triplicate.

### Detection of NO

Nitric oxide (NO) generation was assessed by the Griess method as previously described (34, 35). In brief, the samples were mixed with 100 μl of 1% sulfanilamide, 0.1% N-(1-naphthyl-) ethylenediamine dihydrochloride and 2.5% phosphoric acid. Absorbance was read within 5 min at 550 nm, and the actual concentration was calculated using a standard curve with serial dilutions of sodium nitrite (Molecular Devices).

### ELISA detection

To analyze cytokine production, culture supernatants and tissue homogenates were prepared for cytokine measurement by ELISA. Levels of TNF-α, TGF-β, IFN-γ, IL-12p70, IL-6 and IL-10 in the culture supernatants and sIgA in tissue homogenates were quantified using a sandwich ELISA kit (eBioscience) as per the manufacturer's instructions.

### Flora analysis

Genomic DNA was extracted from the cecal contents using the TIANamp Stool DNA kit as per the manufacturer's instructions. The 16S rDNA v3/4 hypervariable region of the microbial genome was amplified using the primers 341F (5'-CCTACGGGNGGCWGCAG) and 805R (5'-GACTACHVGGGTATCTAATCC). The PCR program was as follows: 1) 94°C for 2 min; 2) 35 cycles of denaturation at 94°C for 20 s, annealing at 52°C for 40 s, and extension at 72°C for 1 min; and 3) 72°C for 2 min followed by cooling at 4°C. The specific label sequence was added to the PCR production in a total volume of 20 μl: 1× reaction buffer (NEB Q5TM), 0.3 mM DNTPs, 0.25 μM F primer, 0.25 μM index primer, 1 U Q5TM DNA polymerase (NEB) and 1 μl of template. The PCR program was as follows: (1) 98°C for 30 s; (2) 11cycles of denaturation at 94°C for 10 s, annealing at 65°C for 30 s, and extension at 72°C for 30 s; and 3) 72°C for 5 min. The PCR product of each sample was recycled, and the DNA concentration was measured using a UV-Vis spectrophotometer (Nano Drop ND1000, USA). Library construction and Illumina MiSeq sequencing were performed at Jing Bai Biotechnology Co., Ltd., (Hangzhou, Zhejiang, China).

### Isolation and identification of BMDCs

Bone marrow-derived dendritic cells (BMDCs) were obtained from healthy C57BL/6 mice as previously reported (36). In brief, cell suspensions were separated from bone marrow cavities in the femur and tibia of the hind legs. After removing tissue clumps and lysing red blood cells, single-cell suspensions were washed, resuspended, and differentiated into BMDCs in the cell culture medium (10% FBS,100 U/ml penicillin, 100 μg/ml streptomycin, and 10 ng/ml M-CSF in RPMI 1640). Cells were collected, and the purity of the CD11c^+^ cells was >90%, as detected by flow cytometry after 6 days of initial BMDC culture.

### Cell cytotoxicity and acid phosphatase assay

Cell viability was estimated using the MTT methods previously reported (35). Briefly, BMDC monolayers were cultured in a 96-well microplate and incubated with GL at different concentrations (0–800 μg/ml) for 48 h. The culture medium was removed, and fresh RPMI1640 containing 0.5 mg/ml MTT was added to each well. After 4 h of incubation, the supernatant was replaced with DMSO. The optical density was measured by SpectraMaxM5 at OD_570_. For the lactate dehydrogenase (LDH) assay, BMDCs were incubated for 48 h with PBS (control), GL (25, 50, 100, 200 μg/ml) and LPS (50 ng/ml). LDH activity in the culture supernatant was measured using the CytoTox96 kit (Roche Diagnostics, Mannheim, Germany) as previously described (37). For the acid phosphatase (ACP) assay, BMDCs were seeded at a density of 5 × 10^5^ cell/well and incubated with PBS (control), GL (25, 50, 100, 200 μg/ml) and LPS (50 ng/ml) for 48 h. The medium was discarded, and the cells were lysed with 0.5% Triton X-100. ACP activity was detected using the acid phosphatase assay kit (Sigma-Aldrich, CS0740) as per the manufacturer's instructions.

### Flow cytometry analysis

Cell suspensions were collected and blocked with 5% normal goat serum for 1 h at 4°C and then incubated with the corresponding Abs (FITC-Toll-like receptor (TLR)-2, -CD40, -CD80, -CD83, -CD86 and -MHC-II) for 1 h at 4°C. After washing, the cells were detected, and the fluorescence signals were determined using a FACScan flow cytometer (BD Biosciences) at 488 nm. At least 10,000 events were collected from the myeloid gate (38).

### Real-time PCR for expression analysis

Total RNA was extracted from BMDCs (RNAiso plus, TAKARA) exposed to different treatments. The transcripts of detected genes were examined and analyzed using quantitative real-time PCR on an ABI 7500 real-time PCR system (Applied Biosystems) using the PrimeScript II 1st Strand cDNA Synthesis Kit (TAKARA). Total RNA was analyzed on a 1.5% (w/v) agarose gel and reverse transcribed into cDNA using the PrimeScript II 1st Strand cDNA Synthesis Kit (TAKARA). All real-time PCRs were performed in a total volume of 10 μl using SYBR Premix Ex Taq II (TAKARA). The PCR program was designed according a previously described protocol (39). The primers used for this experiment are shown in Supplemental Table S1. The results were normalized to β-actin expression, and relative quantification was calculated using the 2^−ΔΔ*Ct*^ method. The sample was run in triplicate parallel reactions in all cases.

### Determination of virulence genes

The expression of virulence genes was determined by RT-qPCR. ST (1 × 10^8^ CFU/mL) were resuspended in Luria-Bcrtani (LB) medium and incubated with PBS, GL (100 μg/mL) or GM (25 μg/mL) at 37°C, respectively. Total RNA was extracted at 0, 1, 2 and 4 h, and then virulence genes (*ssrB, sipB, hilA, invA* and *sopD*) expression were measured by real-time PCR. The primers sequences were listed in Supplemental Table S1. The relative quantification of genes was determined by changes in expression of transcripts relative to expression in untreated ST. Samples were normalized to the reference gene 16s rRNA. Data are mean ± standard deviation for three independent experiments.

### Western blot analysis

All the BMDC samples were lysed in RIPA buffer (Beyotime) on ice for 30 min, and equal amounts of protein lysates were subjected to SDS-PAGE. The proteins were then transferred to nitrocellulose (NC) membranes, which were blocked in blocking solution (Sangon Biotech). After washing, each NC membrane was incubated with the corresponding primary antibody (phospho-JNK, JNK, phospho-p38, p38, p65, IκBα, LaminB1, p-ERK and ERK) overnight at 4°C. Following incubation with the secondary antibody linked to HRP, the bands were visualized with an ECL detection system as per the manufacturer's instructions.

### Statistical analyses

The results are presented as the mean ± standard deviation of three independent experiments. Statistical analyses were performed by applying the two-tailed Student's *t*-test and one-way ANOVA using SPSS 20.0 statistical software (SPSSInc., Chicago, IL, USA). Statistical significance was considered at *P* < 0.05 or *P* < 0.01.

## Results

### GL effectively ameliorates the weight loss of ST-infected mice

**Figure 1:**
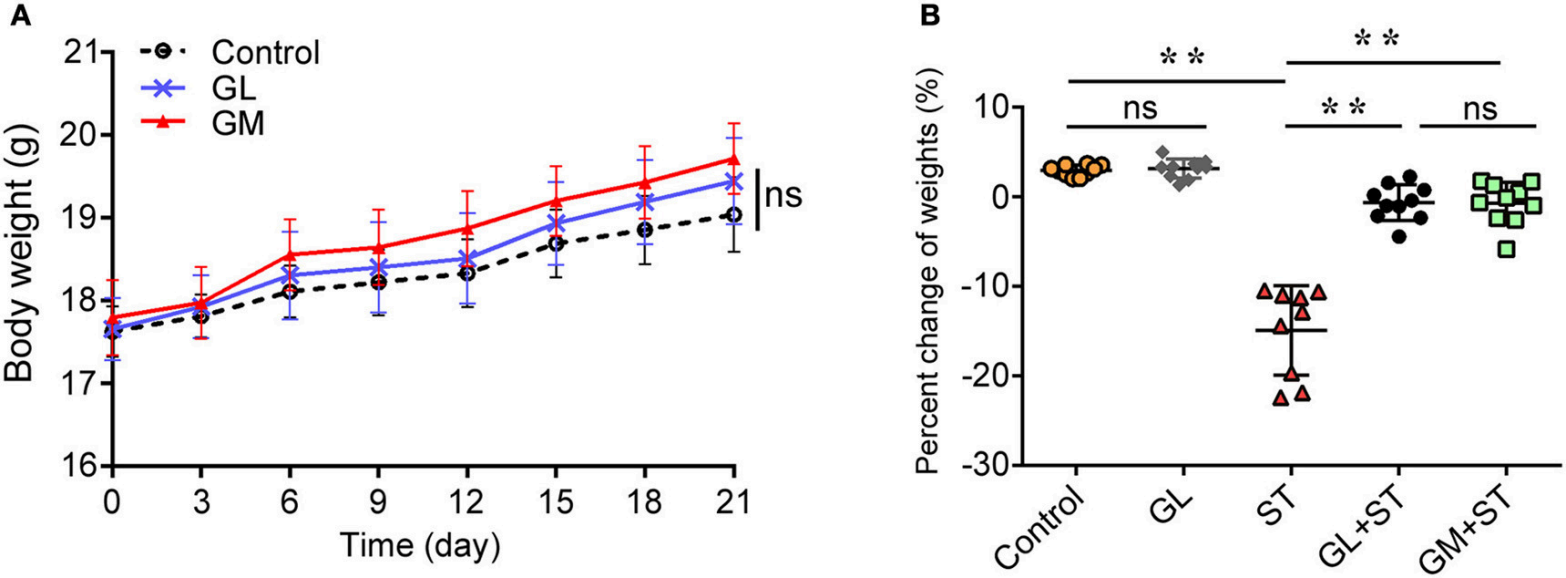
Effect of GL on the body weight of normal and ST-infected mice. **(A)** GL and GM were administered to mice and body weight was monitored every 3 days over 21 days. PBS served as a negative control. **(B)** Mice were infected orally with 4 × 10^8^ CFU of *Salmonella*, except for the control and GL-alone groups. At 72 h post-infection, the percent changes of weight were measured. Significance was analyzed using a paired *t*-test for body weight (*n* = 10/group; **P < 0.01 were accepted as statistically significant; ns, not significant).

Figure 1A showed that GL treatment alone promoted an increase in the body weights of mice within 21 days, but there was no significant difference compared with the body weights of the control mice (*P* > 0.05). However, the GM-alone group showed a significant increase in daily weight gain (*P* < 0.05). After ST infection, body weight was significantly decreased by 16.20% (*P* < 0.01). GL plus ST significantly reduced the weight loss of the infected mice (decreased by only 4.2% compared with that of the GL group, *P* > 0.05; increased by 15.35% compared with that of the ST-infection group, *P* < 0.01), while the GL plus ST group showed no significant difference compared with the GM plus ST group (*P* > 0.05; Figure 1B). These results suggest that GL can effectively reduce the weight loss of mice infected with *Salmonella*.

### GL ameliorates ST-induced intestinal mucosal injury

**Figure 2:**
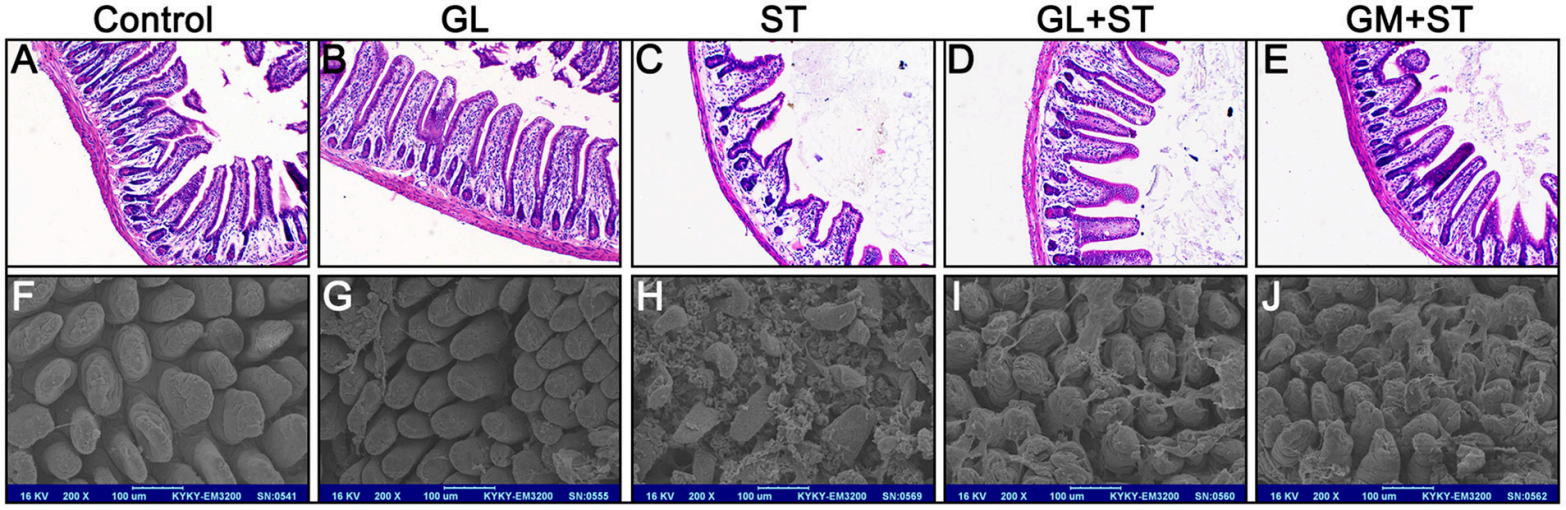
GL attenuates the development of ST-induced ileal mucosal injury in mice. Mice were randomly divided into five groups: Control, GL, ST infection, GL plus ST (GL prevention group) and GM plus ST (GM prevention group). All the mice were infected orally with 4 × 10^8^ CFU of ST except the control and GL groups. After 72 h of infection, the histopathology of the intestinal mucosa was analyzed by H&E staining **(A–E)** and SEM **(F–J)**. Original magnification 100 × **(A–E)**, Scale bar, 100 μm **(F–J)**, *n* = 10.

H&E staining showed that the GL-alone group exhibited an integrated structure of the ileal mucosa, ordered intestinal villi, deep crypts, and a clear and complete gland structure, as also observed in the control group (Figures 2A, B). However, the ST-treatment group showed that: the structure of the ileal mucosa was incomplete, villi were sparse and had a shorter length, crypts were shallow and had a sparse distribution (Figure 2C). With GL plus ST treatment, the intestinal mucosal structure was significantly improved, intestinal villi showed a dense arrangement, and crypts were deep glandular (Figure 2D). Moreover, GM plus ST had an effect similar to that of the GL plus ST treatment (Figure 2E). We utilized SEM to examine the intestinal structure after the different treatments. The results showed that the control group and the GL group had complete ileal villi, which formed full and closely arranged structures (Figures 2F,G). Not surprisingly, the ileal villi in the ST group were severely damaged (Figure 2H), whereas those in both the GL plus ST group and the GM plus ST group showed greater improvement (Figures 2I,J). These observations suggest that GL effectively prevents the ileal mucosal injury caused by ST infection.

**Figure 3:**
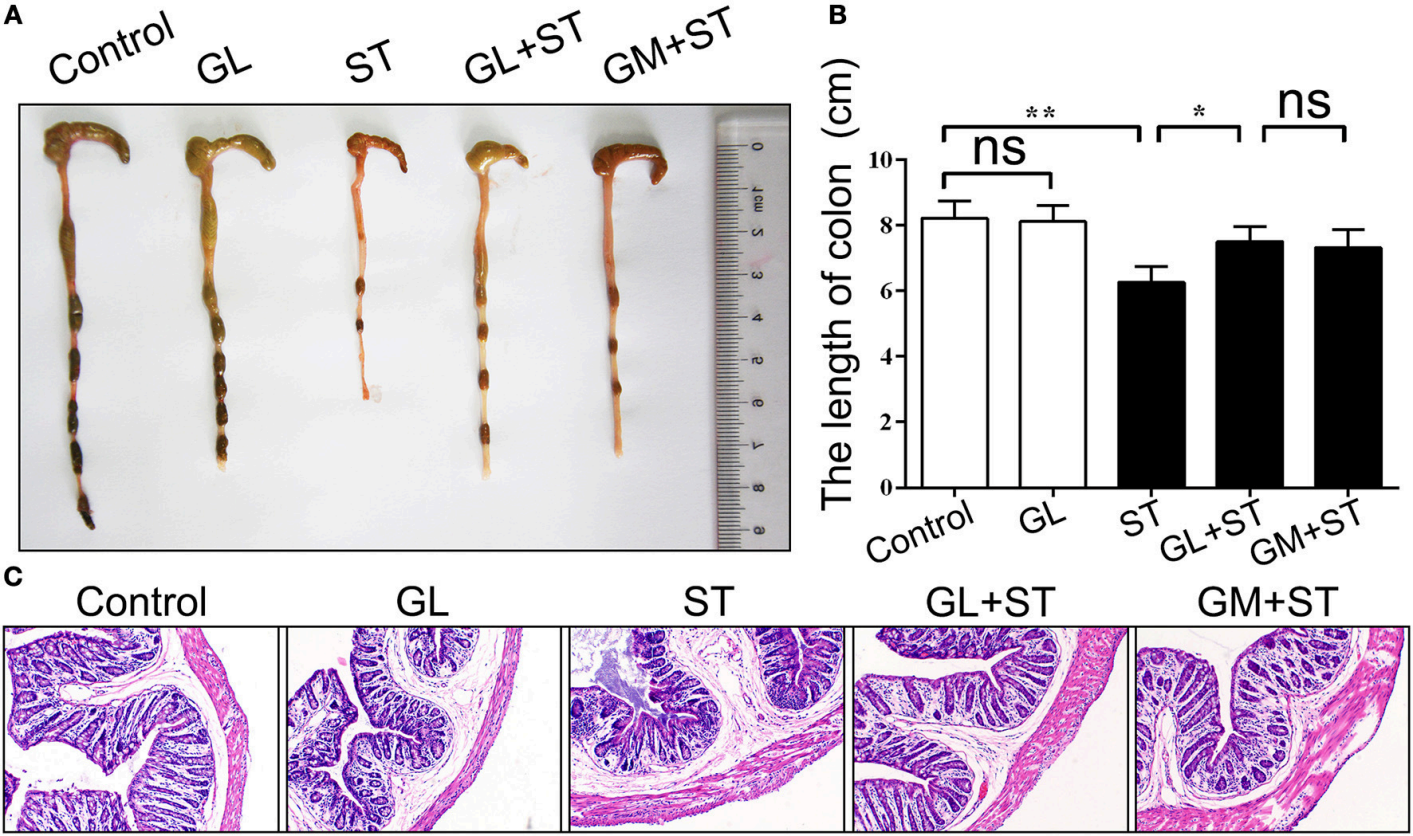
GL administration attenuates cecal and colon damage in ST-infected mice. The mice were randomly divided into five groups: Control, GL, ST infection, GL plus ST (GL prevention group) and GM plus ST (GM prevention group). The mice in the last three groups were infected orally with 4 × 10^8^ CFU of ST and sacrificed 72 h post-infection. Histomorphology **(A)** and length **(B)** of the cecum and colon were observed and counted (*n* = 10/group; *P < 0.05; **P < 0.01), respectively. **(C)** Representative H&E-stained colon sections from each group of mice (original magnification, 100×).

The effect of GL on histopathological changes in the cecum and colon after ST infection were also examined. We found that GL can ameliorates ST-induced cecum and colon injury (Figure 3A). The length of the colon in the control group was 8.21 ± 0.53 cm, and there was no significant difference in the average length of colon between the GL group and the control group (*P* > 0.05). As expected, the length of the colon significantly increased from 6.26 ± 0.48 cm (ST group) to 7.51 ± 0.44 cm (GL plus ST) and 7.32 ± 0.56 cm (GM plus ST; Figure 3B). As shown in Figure 3C, the colonic mucosae in both the control group and GL group were intact, the gland structures were clear, and the mucosal and submucosal cells had no inflammatory cell aggregation. The structure of the colonic mucosa in the ST group was incomplete; however, the intestinal mucosae in the GL plus ST group and the GM plus ST group were improved with a clear reduction in mucosal and submucosal inflammatory aggregation (Figure 3C). These findings suggest that GL can effectively protect the cecum and colon from damage caused by ST infection.

### GL had no direct inhibitory impact on ST growth and virulence gene expression

**Figure 4:**
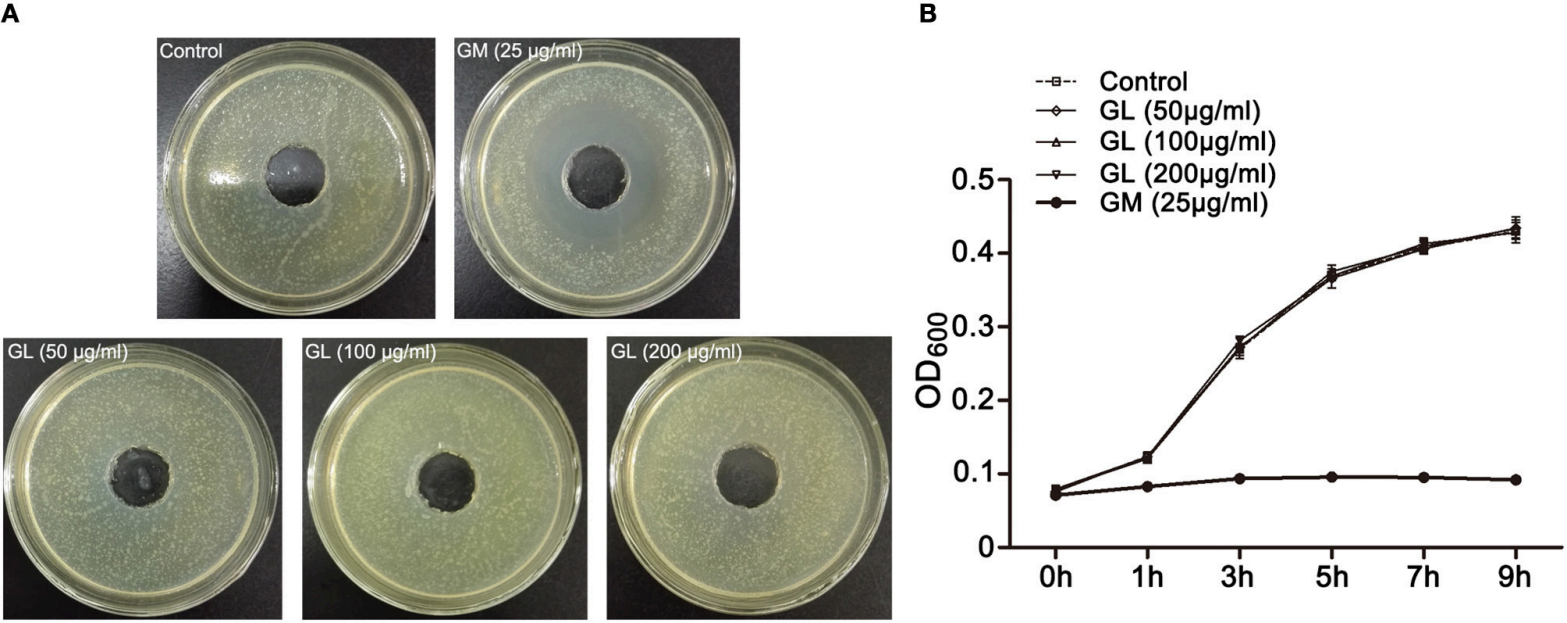
*In vitro* antibacterial activity of GL against ST. **(A)** ST (1 × 10^8^ CFU/mL) were cultured in LB agar medium and treated with various concentrations of GL (0, 50, 100, 200 μg/ml), and GM (25 μg/ml) was used as a positive control at 37°C. The size of the inhibition zone was observed **(B)** and analyzed statistically with a line chart. The OD600 kinetics were determined to analyze the effect of GL against ST.

Because GL had a protective effect on ST-infected mice, we investigated whether it had the ability to inhibit ST growth. An *in vitro* bacterial inhibition assay was performed. We found that the control group and three different concentrations (50, 100, 200 μg/mL) of GL had no significant inhibitory effects (*P* > 0.05), whereas 25 μg/mL GM treatment inhibited ST growth (*P* < 0.05; Figures 4A,B). The above results show that GL has no direct effect on ST growth and proliferation *in vitro*.

We further examined the effects of GL on the expression of ST virulence genes. The virulence genes *invA, sopD, sipB, hilA* and *ssrB* play a key role in ST virulence, and GL had no significant impact on the expression of these genes after co-culture for 1, 2 and 4 h (*P* > 0.05). In contrast, GM downregulated *invA* and *sopD* expression at 1 h (*P* < 0.01). Surprisingly, the expression of *sipB* (*P* < 0.05), *hilA* (*P* < 0.01) and *ssrB* (*P* < 0.01) dramatically increased after co-culture for 4 h (Supplemental Figure S2A). These results suggest that GL can not inhibit the expression of the ST virulence genes *in vitro*.

### Effects of GL on intestinal colonization and translocation of ST in mice

**Figure 5:**
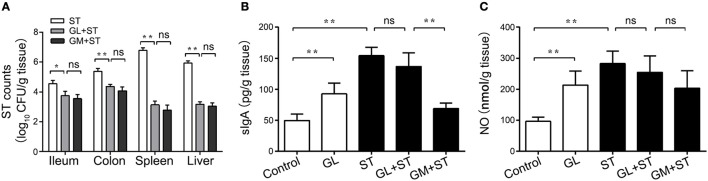
GL inhibits ST colonization, translocation and cytokine secretion in mice. The mice were pretreated with GL and GM, and PBS served as the negative control. The colonization of ST in ileum, colon, liver and spleen was detected after 72 h of ST infection **(A)**. The mice were infected orally with GL, ST infection, GL plus ST (GL prevention group) and GM plus ST (GM prevention group), and PBS served as a negative control. After 72 h of infection, sIgA **(B)** and NO **(C)** secretion in the ileac mucosa were quantified by ELISA, and the data are expressed as the mean ± SD (*n* = 5/group; ns, not significant; *P < 0.05; **P < 0.01).

ST colonization and translocation in the intestine of mice directly determines ST survival and pathogenicity. Therefore, we examined ST levels in the ileum, colon, spleen and liver and found that GL could regulate ST colonization and translocation in the intestines of mice. The number of ST (log_10_ CFU/g tissue) significantly decreased by 17.39% (*P* < 0.05), 18.74% (*P* < 0.01), 53.86% (*P* < 0.01) and 46.76% (*P* < 0.01), respectively, in the GL plus ST group compared with those in the control group. Similar results were observed in the GM plus ST positive control group (Figure 5A). The above results indicate that GL inhibits ST colonization and translocation in ileum, colon, spleen and liver.

### GL modulates cytokine production against ST infection

sIgA and NO can effectively inhibit the colonization and penetration of pathogenic microorganisms, thus maintaining the balance of the microbiota in the intestine. To explore whether GL could protect the ileal mucosa against ST infection via the anti-infective molecules sIgA and NO, detection of sIgA and the NO expression assay were performed. We found that the levels of sIgA and NO in the ileal mucosa dramatically rose to 96.21 and 120.85%, respectively (*P* < 0.01), in the GL-treatment group compared with those in the normal mouse control group. In the ST-treatment group, the production of sIgA and NO increased 215.23 and 192.51%, respectively (*P* < 0.01). The production of sIgA and NO in the ileum of the GL plus ST group was lower than that in the ST group, but this difference was not significant (*P* > 0.05; Figures 5B,C). These findings suggest that GL promotes sIgA and NO secretion against ST infection in the ileal mucosa.

**Figure 6:**
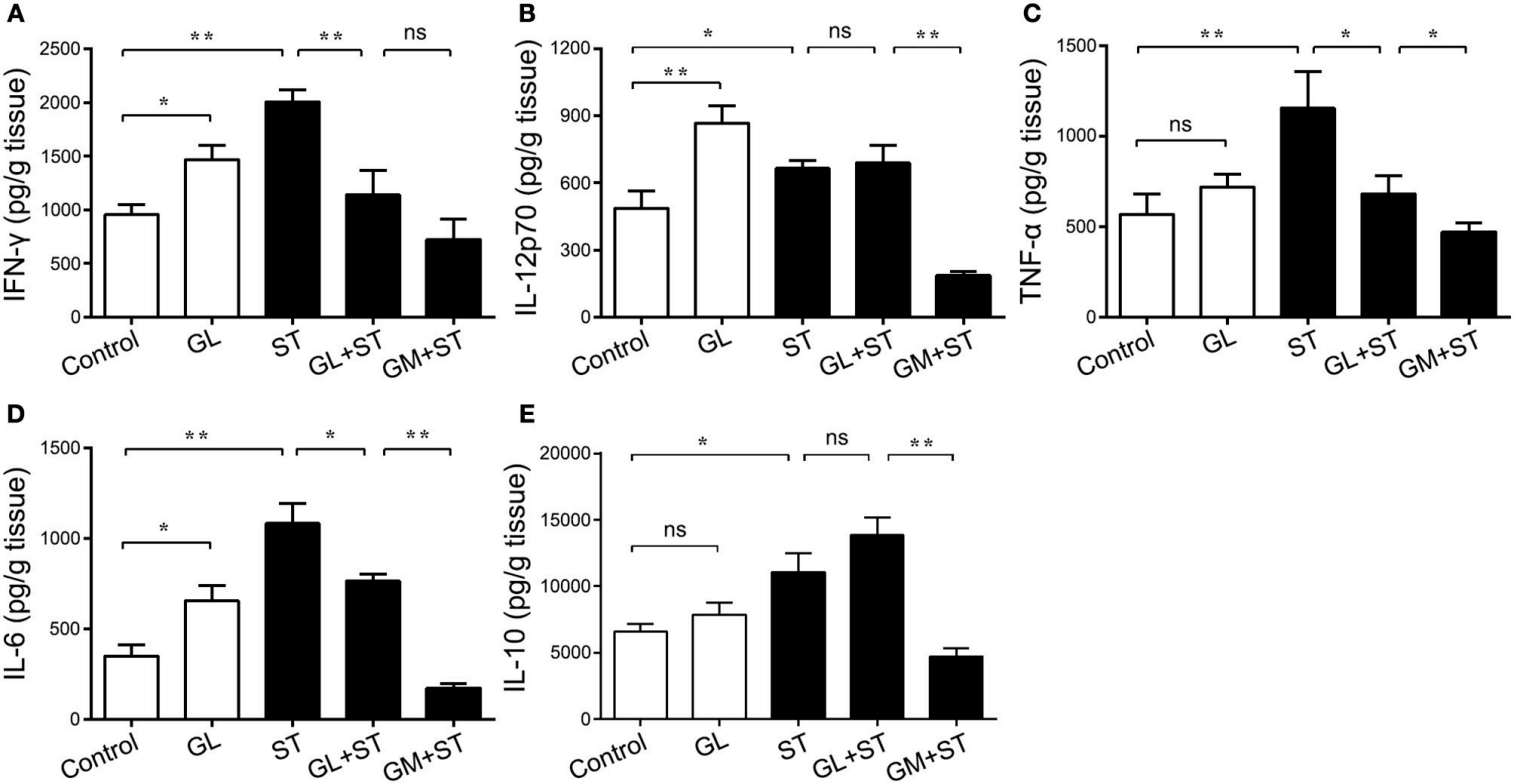
Effect of GL on ileac cytokine secretion in normal and ST-infected mice. The mice were infected orally with GL, ST, GL plus ST (glycyrrhizin prevention group) and GM plus ST (gentamicin prevention group). Body weight was monitored every 3 days over 21 days. PBS served as a negative control. After 72 h of infection, IFN-γ **(A)**, IL-12p70 **(B)**, TNF-α **(C)**, IL-6 **(D)** and IL-10 **(E)** secretion in the ileum were quantified by ELISA, and the data are expressed as the mean ± SD (*n* = 5/group; ns, not significant; *P < 0.05; **P < 0.01).

Additionally, the secretion of the proinflammatory cytokines IFN-γ, IL-12p70 and IL-6 in the ileal mucosa significantly increased by 53.92% (*P* < 0.05), 78.33% (*P* < 0.01) and 87.49% (*P* < 0.05) in the GL group compared with that in the control group (Figures 6A,B,D). In contrast, the secretion of TNF-α and IL-10 showed an upward trend, but the effect was not significant (*P* > 0.05; Figures 6C,E). As expected, the secretion of IFN-γ, IL-12p70, TNF-α, IL-6, and IL-10 increased considerably by 109.98% (*P* < 0.01), 36.57% (*P* < 0.05), 103.35% (*P* < 0.01), 210.15% (*P* < 0.01) and 67.72% (*P* < 0.05), respectively, in the ST group compared with that in the control group. The secretion of IFN-γ, TNF-α and IL-6 in the GL plus ST group significantly decreased by 76.28% (*P* < 0.01), 40.86% (*P* < 0.05) and 29.51% (*P* < 0.05), respectively (Figures 6A–D), compared with that in the ST group. The above results indicate that GL improves the anti-infective response by increasing the levels of the proinflammatory cytokines IFN-γ, IL-12p70 and IL-6 in mice.

**Figure 7:**
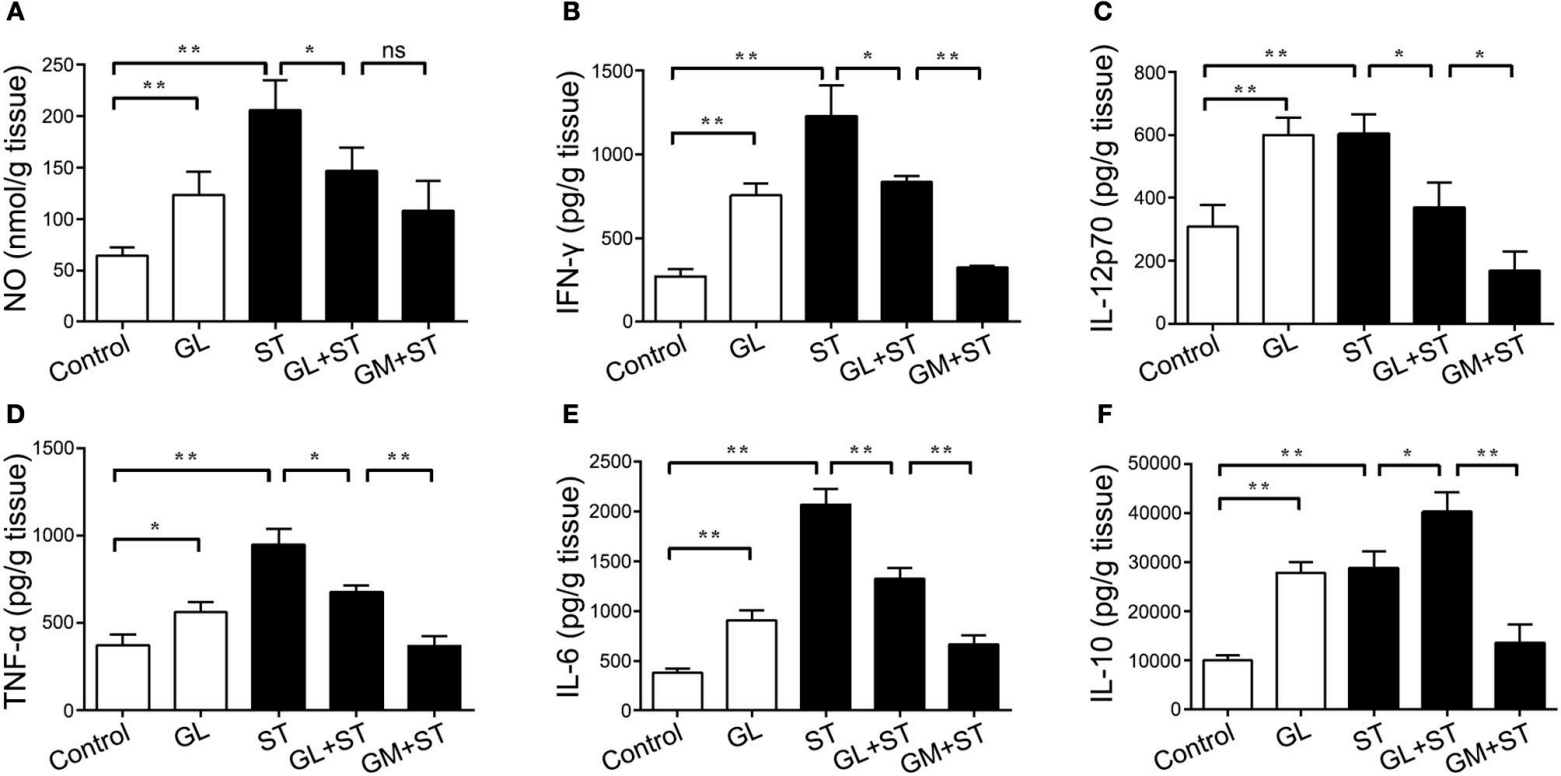
Effect of GL on colonic cytokine secretion in normal and ST-infected mice. The mice were infected orally with GL, ST, GL plus ST (glycyrrhizin prevention group), or GM plus ST (gentamicin prevention group). After 72 h of infection, body weight was monitored every 3 days over 21 days. PBS served as a negative control. NO **(A)**, IFN-γ **(B)**, IL-12p70 **(C)**, TNF-α **(D)**, IL-6 **(E)** and IL-10 **(F)** secretion in colon were quantified by ELISA, and the data are expressed as the mean ± SD (*n* = 5/group; ns, not significant; *P < 0.05; **P < 0.01).

Furthermore, we examined the expression of cytokines in the colon. The administration of GL significantly upregulated the expression levels of the inflammation-related cytokines NO (*P* < 0.01), IFN-γ (*P* < 0.01), IL-12p70 (*P* < 0.01), TNF-α (*P* < 0.05), IL-6 (*P* < 0.01) and IL-10 (*P* < 0.01; Figures 7A–F). Moreover, these inflammatory factors in the ST group showed similar results, whereas pretreatment with GL significantly reduced the production of IFN-γ (*P* < 0.05), IL-12p70 (*P* < 0.05), TNF-α (*P* < 0.05), IL-6 (*P* < 0.01), and NO (*P* < 0.05) in ST-infected mice (Figures 7A–E). As expected, the secretion of anti-inflammatory cytokine IL-10 (*P* < 0.05) in the GL plus ST group was also promoted to avoid excessive inflammation in the colon (Figure 7F), compared with that in the ST group. These findings suggest that GL promotes the secretion of inflammation-related cytokines against ST infection.

### GL increases the levels of proinflammatory cytokines in serum

**Figure 8:**
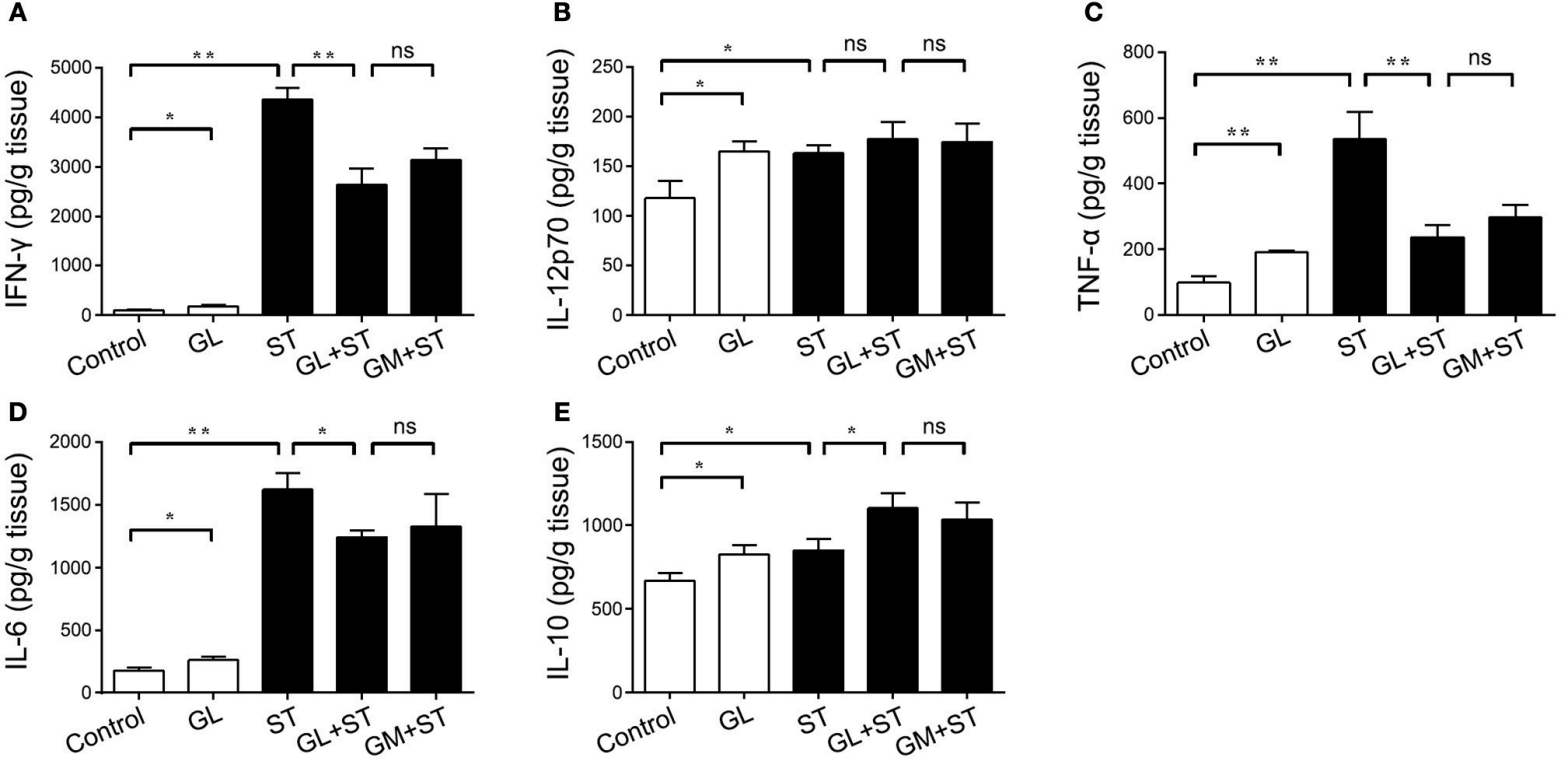
Effect of GL on serum cytokine secretion in normal and ST-infected mice. The mice were infected orally with GL, ST, GL plus ST (GL prevention group), or GM plus ST (GM prevention group). After 72 h of infection, body weight was monitored every 3 days over 21 days. PBS served as a negative control. IFN-γ **(A)**, IL-12p70 **(B)**, TNF-α **(C)**, IL-6 **(D)** and IL-10 **(E)** secretion in serum was quantified by ELISA, and the data are expressed as the mean ± SD (*n* = 5/group; ns, not significant; *P < 0.05; **P < 0.01).

Cytokines in the gut can enter blood vessels and play an important role in protecting the gut from ST infection. A cytokine detection assay was performed to elucidate the effect of GL on the levels of proinflammatory cytokines in serum. GL treatment increased the levels of IFN-γ (*P* < 0.05), IL-12p70 (*P* < 0.05), TNF-α (*P* < 0.01), IL-6 (*P* < 0.05) and IL-10 (*P* < 0.05) in the serum of mice compared with those in the control group. After infection with ST, the levels of the inflammatory cytokines IFN-γ (*P* < 0.01), IL-12p70 (*P* < 0.05), TNF-α (*P* < 0.01), IL-6 (*P* < 0.01) and IL-10 (*P* < 0.05) in the serum of the ST group were significantly increased, whereas GL pretreatment reduced the production of IFN-γ (*P* < 0.01), TNF-α (*P* < 0.01) and IL-6 (*P* < 0.05) caused by ST and promoted the secretion of the anti-inflammatory cytokine IL-10 (*P* < 0.05), which is consistent with the results obtained for the ileum and colon (Figures 8A–E). This result indicates that GL promotes the secretion of immune factors in serum to prevent further ST infection.

### The function of GL in the intestinal microbiota

The microbiota play a key role in maintaining intestinal homeostasis and balance. We questioned whether GL could modulate the intestinal microbiota to reverse the intestinal injury in ST-infected mice. We analyzed the differences in the intestinal microbiota in normal mice and ST-infected mice with or without GL pretreatment. The results showed that ST significantly reduced the relative abundance of *Firmicutes* and *Proteobacteria* and significantly increased the relative abundance of *Verrucomicrobia*. However, GL significantly reduced the relative abundance of *Verrucomicrobia* (*P* < 0.01; Supplemental Figure S3). Moreover, the relative abundance of *Verrucomicrobia* was significantly (*P* < 0.01) downregulated, and *Bacteroidetes* was significantly (*P* < 0.05) upregulated in the GL plus ST group. Similar results were obtained for the GM plus ST group (Supplemental Figure S3).

**Figure 9:**
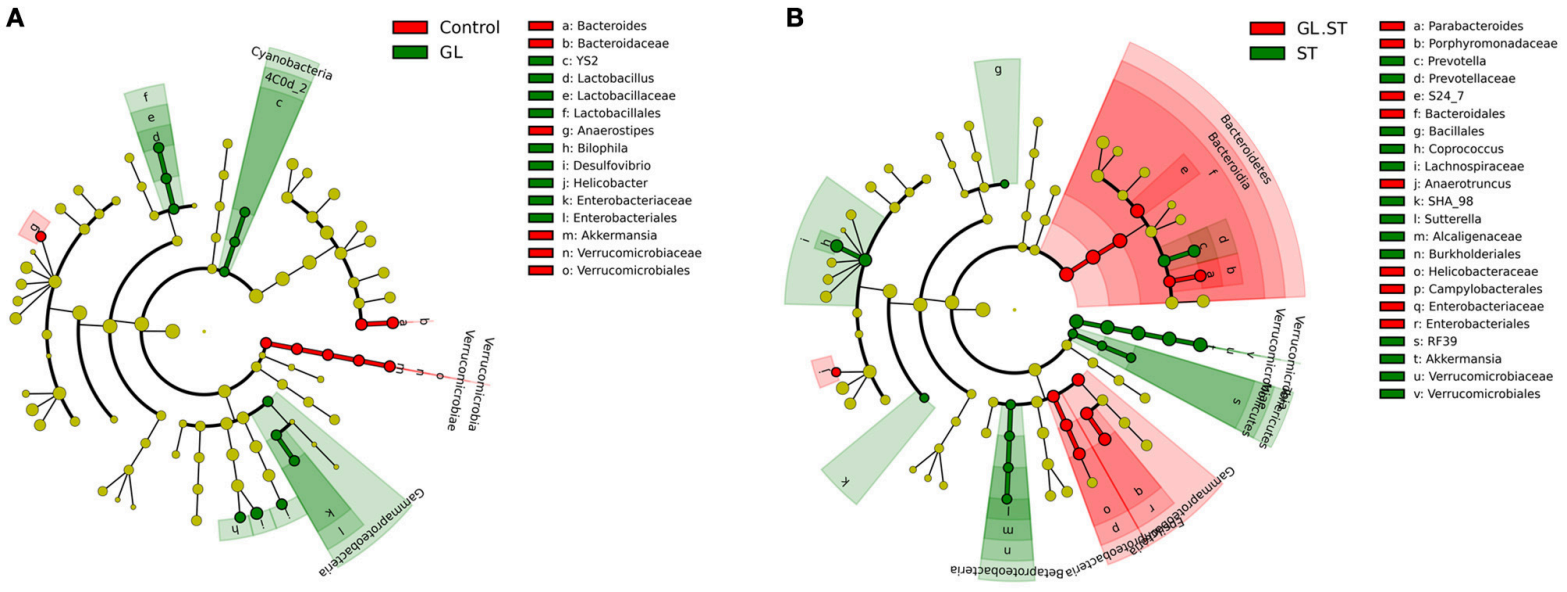
Differences in the gut microbiota in the cecum were determined using the LefSe analytic method. All the samples were evaluated using the LefSe analytic method (*P* < 0.05, log LDA score>2). Comparison of the intestinal microbiota at the genus level between the GL group and control group **(A)** and GL plus ST group and ST group **(B)**. Four replicates were assessed in each group. Control, PBS treatment; GL, glycyrrhizin pretreatment; ST, *S*. Typhimurium infection; GL plus ST, glycyrrhizin pretreatment and *S*. Typhimurium infection.

We further applied the LefSe analytic method to identify differentially abundant bacterial taxa among these groups (only those taxa that obtained a log linear discriminant analysis [LDA] score>2 were ultimately considered). As shown in Figure 9A, the levels of *Cyanobacteria, Lactobacillus, Desulfovibrio, Helicobacter* and *Bilophila* were significantly increased in the cecal microbiota with GL treatment. However, *Verrucomicrobia, Akkermansia, Bacteroides and Anaerostipes* had opposite results. Moreover, the following microbiota decreased in the GL plus ST groups: *Verrucomicrobia, Akkermansia, Sutterella, Prevotella* and *Coprococcus*. The levels of other microbiota (including *Bacteroidetes, Parabacteroides* and *Anaerotruncus*) were improved (Figure 9B). These findings demonstrate that GL modulates the intestinal microbiota, especially *Verrucomicrobia* and *Bacteroidetes*.

### Determination of the non-cytotoxic dose of GL in BMDCs

BMDCs have been considered an important type of antigen-presenting cell (APC) that contributes to the immune response to *Salmonella* (40). Therefore, we hypothesized that GL might inhibit *Salmonella* infection by modulating DC activation and maturation. First, the cytotoxicity of GL to BMDCs was evaluated. Cell viability was determined using the CCK-8 assay. As shown in Supplemental Figure S4A, GL at a range of concentrations (from 12.5 to 400 μ/ml) had no significant effect on BMDC cell activity in mice (*P* > 0.05). Moreover, we measured LDH activity in the cell culture supernatant. There was no significant difference in LDH activity following treatment with GL at a range of concentrations (from 12.5 to 200 μg/ml) compared with that in the untreated control (*P* > 0.05; Supplemental Figure S4B). The above results showed that 200 μg/ml was the optimal concentration, which was used in subsequent experiments.

### Effects of GL on the maturation and antigen presentation of BMDCs

**Figure 10:**
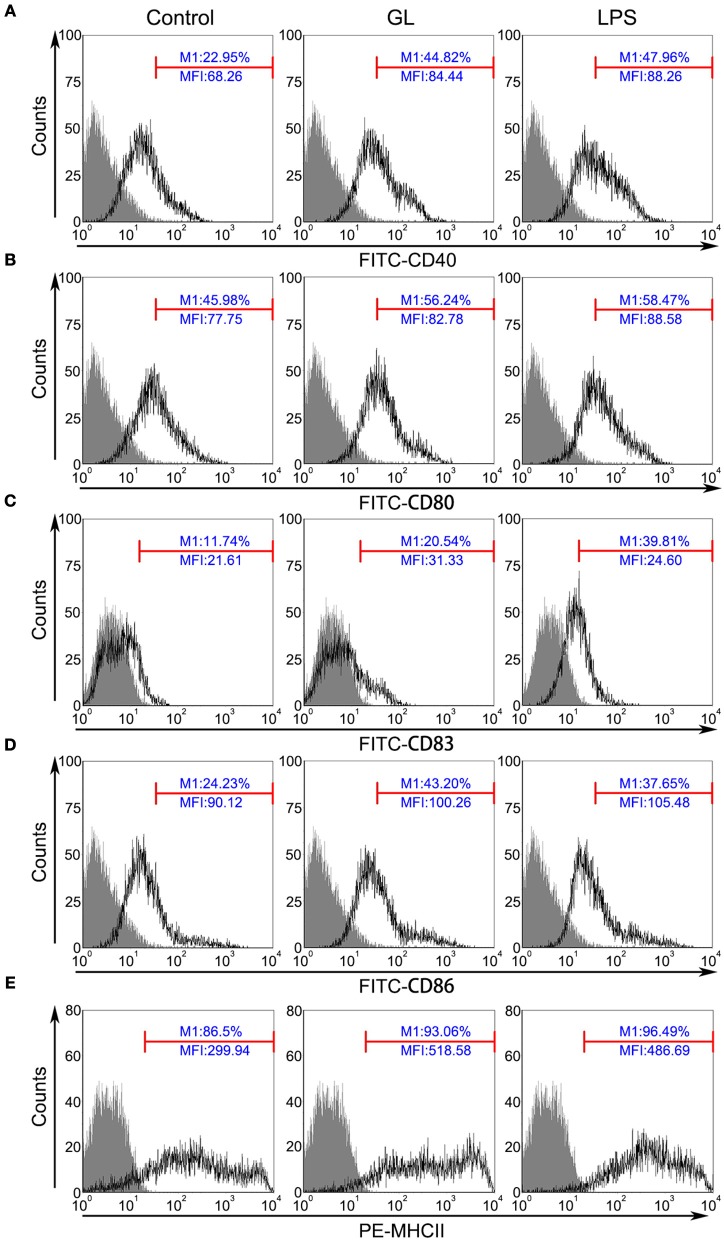
GL improves surface marker expression in BMDCs. BMDCs were treated with PBS (blank control), GL (200 μg/ml), or LPS (50 ng/ml; positive control) for 48 h. Cells were stained with antibodies against CD40 **(A)**, CD80 **(B)**, CD83 **(C)**, CD86 **(D)**, and MHC-II **(E)**, and the fluorescence signals were determined immediately using a FACScan flow cytometer. At least 10,000 events were collected from the cell gate. The data represent three independent experiments.

To further clarify the involvement of GL in mature BMDCs, the key accessory molecules CD40, CD80, CD83, CD86 and MHC-II in BMDCs were assessed by flow cytometry. Typically, these markers are expressed at lower levels in immature DCs, whereas higher expression is detected in mature DCs following activation. As shown in Figure 10, the percentages of CD40, CD80, CD83, CD86 and MHC-II expression were significantly increased by 95.29% (*P* < 0.01), 22.31% (*P* < 0.05), 74.96% (*P* < 0.01), 78.29% (*P* < 0.01) and 7.58% (*P* < 0.05), respectively, in the GL-treated group compared with those in the control group. The above results confirmed that GL could effectively induce BMDC maturation.

ACP is a key enzyme in the lysosomes of BMDCs and participates in antigen presentation. Immature BMDCs have higher ACP activity and a better ability to capture and process antigens. By contrast, intracellular ACP activity in mature BMDCs decreases and antigen presentation capacity increases (41). To evaluate the effect of GL on antigen presentation in BMDCs, we measured the ACP activity in cytoplasmic BMDCs. We found that GL reduced the activity of ACP in a dose-dependent manner (*P* < 0.05). Notably, 200 μg/ml GL significantly downregulated the activity of ACP in BMDCs (*P* < 0.01), which was consistent with the previous cytotoxicity assay (Supplemental Figure S4C). These results provide *in vitro* evidence that GL promotes antigen presentation by inhibiting the activity of ACP in BMDCs.

### Effects of GL on cytokine secretion in BMDCs

**Figure 11:**
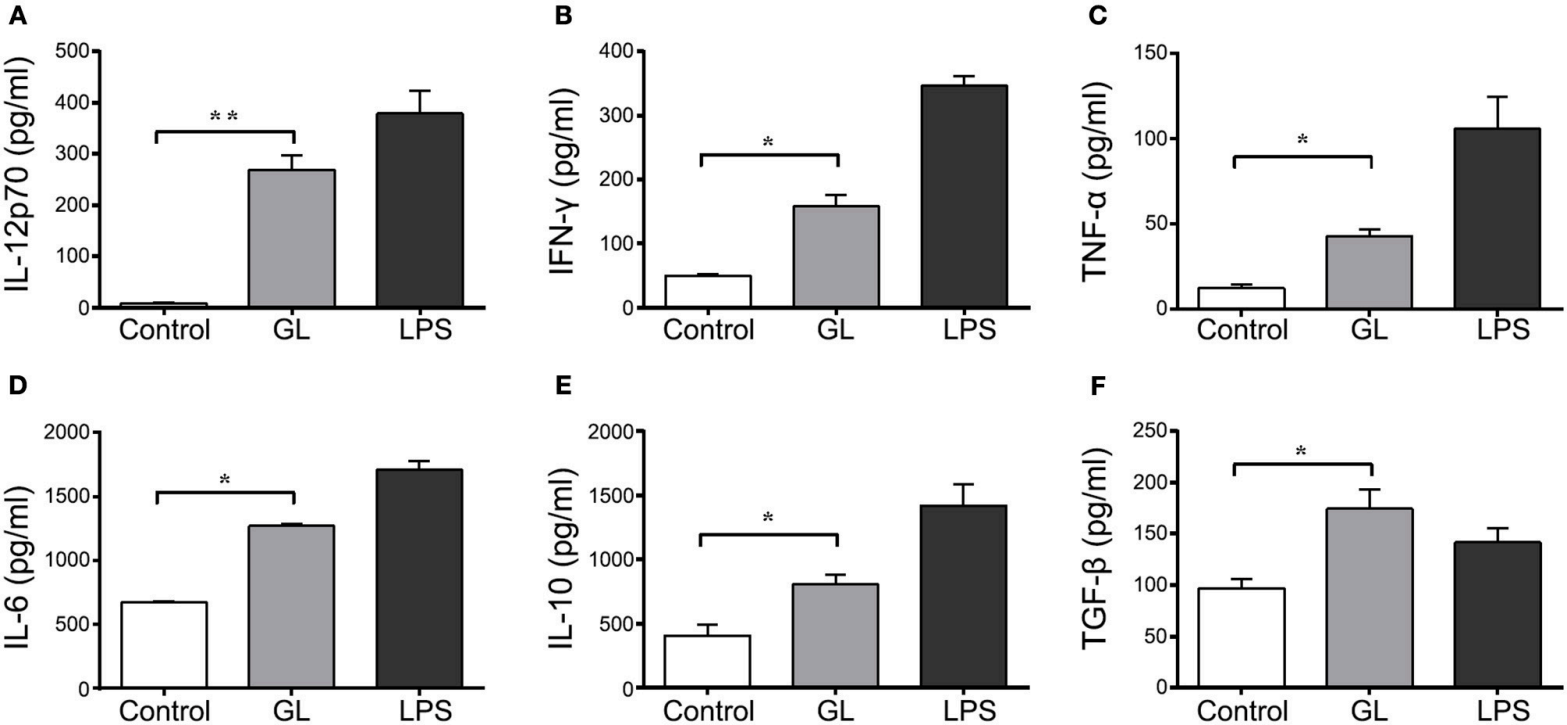
Effect of GL on cytokine secretion in murine BMDCs. BMDCs were co-incubated with PBS, GL (200 μg/ml) and LPS (50 ng/ml); PBS served as a negative control. After 48 h, the cell supernatant was collected. IL-12p70 **(A)**, IFN-γ **(B)**, TNF-α **(C)**, IL-6 **(D)**, IL-10 **(E)** and TGF-β **(F)** secretion in supernatant were quantified by ELISA. The data represent three independent experiments. The mean ± SD of the results from three independent experiments is shown. *P < 0.05; **P < 0.01.

Cytokine secretion in BMDCs after a 48 h treatment with GL was also assessed by ELISA. As shown in Figure 11, the Th1 cytokines IL-12p70 and IFN-γ were significantly elevated in the supernatant of BMDCs treated with GL by 2882.50% (*P* < 0.01) and 215.84% (*P* < 0.05), respectively, compared with those in the control, while the proinflammatory cytokines TNF-α and IL-6 were significantly elevated by 248.81% (*P* < 0.05) and 89% (*P* < 0.05), respectively. In contrast, no differences were observed in the Th2 cytokines IL-4 and IL-13 (*P* > 0.05; data not shown). Furthermore, the anti-inflammatory cytokines IL-10 and TGF-β significantly increased in the GL-treatment group by 98.63% (*P* < 0.05) and 80.76% (*P* < 0.05), respectively, compared with those in the control (Figures 11E,F). These data indicate that GL upregulates the production of IL-12p70, IFN-γ, TNF-α, and IL-6 in BMDCs to enhance Th1 immune response. Moreover, GL can also increase the secretion of the anti-inflammatory cytokines IL-10 and TGF-β to attenuate excessive inflammation and promote the termination of the late inflammatory response.

### Functional evaluation of the effect of GL on tlr expression in BMDCs

TLRs are considered important elements in pathogen recognition and DC maturation (42). To examine whether GL could activate TLR pathways, we detected the gene expression of TLRs (TLR1-5, TLR7, TLR9) and their downstream adaptors (MyD88 and TRIF) in BMDCs treated with GL for 3, 6 and 12 h. As demonstrated in Table 1, *tlr2* expression was significantly upregulated in the GL group at 3, 6 and 12 h by 114% (*P* < 0.05), 158% (*P* < 0.05) and 353% (*P* < 0.01), respectively. By contrast, *tlr3* was significantly downregulated by 48% (*P* < 0.05), 41% (*P* < 0.05) and 41% (*P* < 0.05), respectively. *tlr4* expression decreased by 43% (*P* < 0.05), 16% (*P* > 0.05) and 37% (*P* < 0.05), respectively; however, GL treatment had no significant effect on the expression of *tlr1, tlr5, tlr7, tlr9, myd88* and *trif* at the above three time points (*P* > 0.05). Moreover, the upregulation of GL on TLR2 in BMDCs was further confirmed by FCM. After treatment for 48 h, the mean fluorescence intensity (MFI) of TLR2 on the surface of BMDCs in the GL group was increased by 150.72% compared with that in the control, which was consistent with the results of the gene expression analysis (Supplemental Figure S5). These results implied that GL could increase *TLR2* gene expression to induce the maturation of murine DCs.

**Table 1:** Effects of GL on gene expression of *tlrs, myd88, trif* in BMDCs.

	**Time points of GL treatment**		
**Genes**	**3 h**	**6 h**	**12 h**
*tlr1*	0.91 ± 0.15	1.13 ± 0.18	1.69 ± 0.30
*tlr2*	2.14 ± 0.12^*^	2.58 ± 0.22^*^	4.53 ± 0.57^**^
*tlr3*	0.52 ± 0.08^#^	0.59 ± 0.05^#^	0.59 ± 0.07^#^
*tlr4*	0.57 ± 0.07^#^	0.84 ± 0.14	0.63 ± 0.08^#^
*tlr5*	1.34 ± 0.19	0.94 ± 0.13	1.03 ± 0.23
*tlr7*	0.99 ± 0.13	1.12 ± 0.11	0.95 ± 0.16
*tlr9*	1.01 ± 0.17	1.40 ± 0.16	1.23 ± 0.24
*myd88*	1.14 ± 0.14	0.89 ± 0.11	1.46 ± 0.20
*trif*	1.16 ± 0.18	1.42 ± 0.21	1.30 ± 0.12

*The related gene expression level was calculated using the 2^−ΔΔCT^ method, and the expression of the control group was arbitrarily set at 1.0. The data represent three independent experiments. The mean ± SD of the results from three independent experiments is shown*.

* *significantly higher than the control group (P < 0.05)*;

** *significantly higher than the control group (P < 0.01)*;

# *significantly lower than the control group (P < 0.05)*.

### Functional evaluation of GL in the MAPK and NF-κB signaling pathways in BMDCs

**Figure 12:**
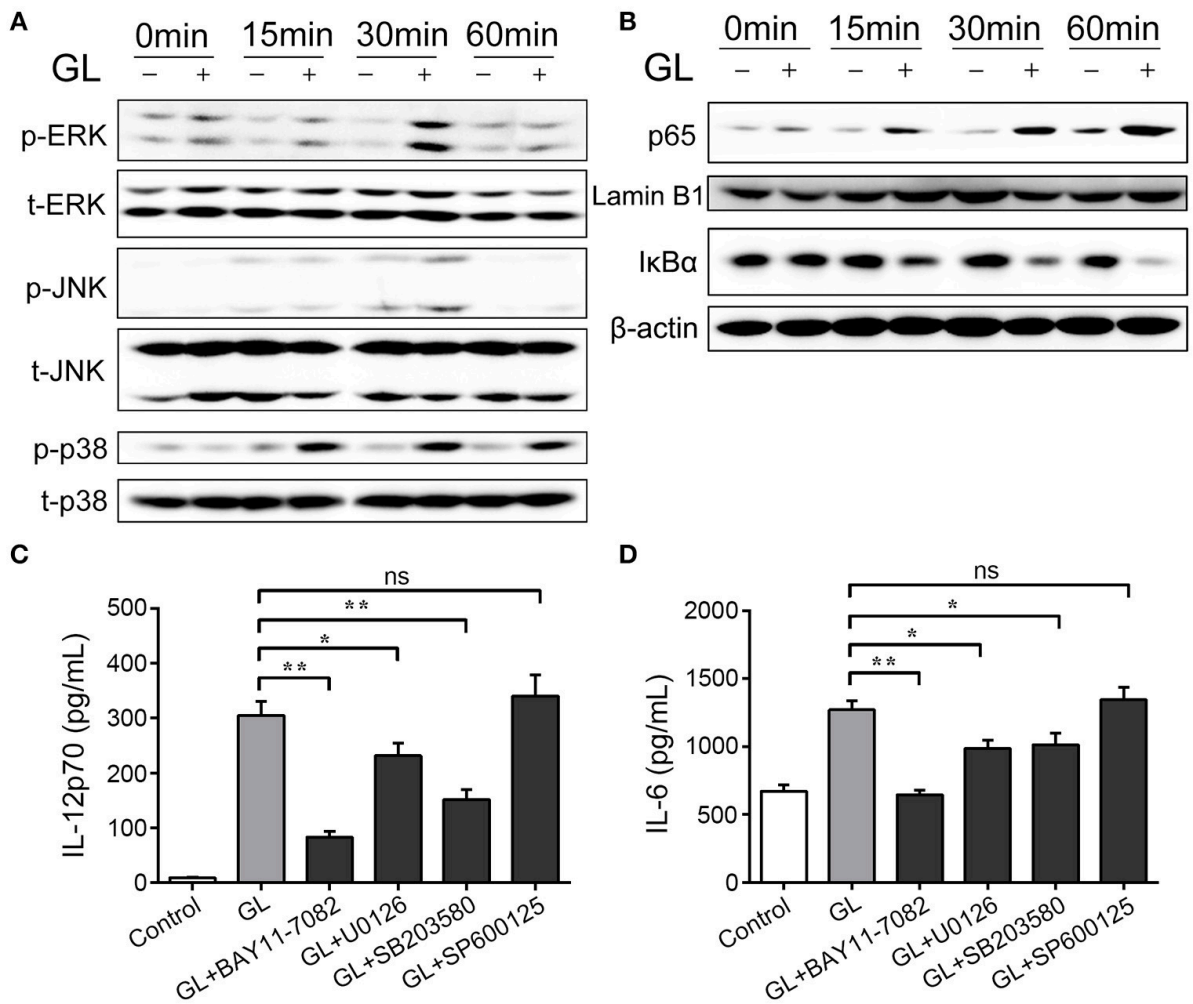
Effect of GL on the MAPK and NF-κB signaling pathways in murine BMDCs. The cells were treated with PBS (-, control) or GL (plus, 200 μg/ml) at the indicated time points. **(A)** Cell lysates were prepared, and phosphorylation of total (t)-ERK, phosphorylated (p)-ERK, t-JNK, p-JNK, t-p38 and p-p38ERK1/2 was analyzed by western blotting. **(B)** Nuclear and cytosolic extracts in BMDCs were prepared and detected with p65 and IκBα antibody by western blotting. **(C, D)** BMDCs were pretreated with NF-κB inhibitor (BAY 11-7082, 20 μM), JNK inhibitor (SP600125, 20 μM), p38 MAPK inhibitor (SB203580, 20 μM), or ERK1/2 inhibitor (U0126, 20 μM), followed by treatment with GL (200 μg/ml) for 30 min, and then IL-12p70 and IL-6 secretion in supernatant were quantified by ELISA. The data represent three independent experiments. The mean ± SD of the results from three independent experiments is shown. *P < 0.05; **P < 0.01.

Both the MAPK and NF-κB signaling pathways participate in the regulation of DC maturation (43). Therefore, the phosphorylation levels of MAPKs (ERK, JNK and p38) and the nuclear translocation of NF-κB subunit p65 were assessed by western blot analysis at different time points. As shown in Figure 12A, the levels of phosphorylation of ERK and p38 MAPK could be dynamically monitored at 0, 15, 30 and 60 min. The phosphorylation intensity peaked after 30 min of GL treatment and then declined to basal levels within 60 min. Moreover, only weak phosphorylation of JNK was detected at 30 min compared with the control. Furthermore, GL triggered a marked increase in NF-κB p65 levels in the nucleus of BMDCs in a time-dependent manner (Figure 12B), and IκBα protein (a potent inhibitor of NF-κB) gradually decreased in the cytoplasm within 60 min after GL stimulation.

To further validate the involvement of the NF-κB and MAPK signaling pathways in GL-induced BMDC maturation, inhibitor assays were performed. BMDCs were pretreated with specific inhibitors: BAY11-0782 (NF-κB inhibitor); SP600125 (JNK inhibitor); U0126 (ERK1/2 inhibitor); and SB203580 (p38 inhibitor). Pretreatment with the NF-κB inhibitor, the ERK1/2 inhibitor or the p38 inhibitor significantly decreased GL-induced IL-12p70 and IL-6 secretion in BMDCs, while JNK inhibition did not alter IL-12p70 and IL-6 production (Figures 12C,D). These results support the idea that GL induces BMDC maturation though the ERK, p38 MAPK and NF-κB signaling pathways but not the JNK signaling pathway.

## Discussion

GL has a variety of biological activities and can be used as an additive in food as an immunomodulator for the prevention and treatment of diseases (44). Considerable research has shown that natural triterpene saponins extracted from plants have antibacterial (bacterial and fungal), antiviral and antiparasitic activities (45, 46). At present, most research has focused on the antiviral activity of GL, and therefore, the antibacterial ability of GL remains unclear. GL has selective antibacterial ability and significant cytotoxic activity against the streptococcal mutant UA159 but no significant effect on the growth of penicillin-resistant *Staphylococcus aureus* and *Candida albicans* (11, 12). *Salmonella* is one of the major food sources of pathogens (25). *S*. Typhimurium pathogenesis depends mainly on the gene expression of two pathogenicity islands (SPI1and SPI2), and SPI1 is essential for the penetration of intestinal epithelium. ST enters the intestine and adheres to the surfaces of intestinal (mainly ileum and colon) epithelial cells to subsequently arrive in the subepithelial tissue via a series of invasive pathological pathways (47, 48). In the current study, we found that GL could relieve ST-induced weight loss and intestinal mucosal injury by H&E staining and SEM examination. The antibacterial activity assay revealed GL could not directly inhibit ST growth, and real-time PCR showed that GL failed to influence the expression of virulence genes (*invA, hilA, sipB, sopD* and *ssrB*). Studies have shown that the number of ST in a tissue directly reflects the status of infection and the ability of the tissue to resist infection (49). The bacterial colonization assay showed that GL pretreatment significantly reduced the number of ST in mouse ileum and colon. We have provided evidence that GL could effectively inhibit the colonization of ST in the intestine but could not directly inhibit ST growth and virulence gene expression.

To further explore the mechanism of GL in protection against intestinal mucosal injury in mice, we analyzed intestinal mucosal immunity under different conditions and found that GL significantly increased the secretion of NO and sIgA in the intestinal mucosa. The anti-infective effects of the molecules NO, sIgA and IFN-γ have a strong bactericidal effect in the early stage of bacterial infection inhibition (50–52). NO is a key effector that eliminates invading pathogens, especially intracellular pathogens. Moreover, it has strong bactericidal effects together with reactive oxygen species (ROS) (53, 54). sIgA, produced mainly by small intestinal mucosal plasma cells, is an important effector molecule of the intestinal mucosal-specific immune response and serves as the first line of defense against pathogens that have invaded the mucosa (55, 56). Furthermore, GL treatment upregulated the secretion of IFN-γ, IL-12p70 and IL-6 in the ileum, colon and serum. Taken together, these findings imply that GL activates intestinal non-specific immunity to improve the anti-infective ability of the intestine against ST infection.

The intestinal flora plays an important role in combating *Salmonella* invasion and maintaining intestinal immunity (57, 58). The present study showed that ST infection significantly increased the relative abundance of *Verrucomicrobia* and *Akkermansia* in the cecum. Stecher et al. obtained similar results in an intestinal microbiota analysis. The authors noted that ST induces intestinal inflammation and changes the flora of the intestine. The relative abundance of the *Verrucomicrobia* is significantly increased after ST infection (57). Ganesh reported that the relative abundance of *Akkermansia* positively correlates with intestinal inflammation in a murine chronic enteritis model, and *Akkermansia* significantly increases the ST-induced gut inflammatory response (57). In the present study, GL pretreatment significantly reduced the relative abundance of *Verrucomicrobia* and attenuated the intestinal inflammatory response in ST-infected mice, which is consistent with the results of the previous study. Notably, GL alone also significantly reduced the relative abundance of *Verrucomicrobia* and *Akkermansia* in the cecum. These findings indicated that GL can also regulate specific intestinal flora against ST infection.

The main feature of DC maturation is the abatement of antigen uptake and the enhancement of antigen presentation. ACP is the key enzyme in digestion and antigen processing in DCs, and its activity decreases, followed by the maturation of DCs (24). Previous research has shown that GL has the capacity to upregulate the allostimulatory activity of mouse splenic DCs along with the expression of CD40, CD86, and MHC-capital I maturation markers on DCs (24). Similarly, GL induces phenotypic maturation with increased expression of CD86, CD40, CD80, CD83, and MHC-II and decreases the activity of ACP. However, the mechanism responsible for its positive modulation remains obscure. We found that GL treatment significantly upregulated the expression of TLR2 (mRNA and protein) and downregulated the gene expression of *tlr3* and *tlr4* in BMDCs, yet it had no effect on the gene expression of *tlr1, tlr5, tlr7, tlr9, myd88* and *trif*. TLR2 can widely recognize lipoproteins of various pathogens, peptidoglycans of Gram-positive bacteria, and some atypical LPS (59), while TLR2 can also interact with other TLRs, such as TLR1, TLR4, TLR6 (60). GL significantly up-regulated the expression of TLR2 in BMDCs, suggesting that GL can enhance the ability of BMDCs to recognize the above ligands, thereby amplify the receptor signal of TLRs during infection. TLR3 mainly recognizes double-stranded RNA (61). Studies have shown that the expression level of TLR3 is closely related to the maturation status of DC: XCR1^+^cDC activated by PRRs signal during maturation will down-regulate the expression of TLR3 (62). Thus, the down-regulation of TLR3 in BMDCs caused by GL may reflect the maturation state of the cells. TLR4 mainly recognizes LPS of Gram-negative bacteria. GL treatment decreases the expression of TLR4 in BMDCs, which may result in decreased sensitivity of cells to LPS. It indicated that TLR4 receptor signal has been activated, because LPS treatment also down-regulates TLR4 expression, causing tolerance (63). It is not yet known how BMDCs recognize GL, but changes in the expression of these TLRs suggest that GL processing may affect TLRs in BMDCs. MAPKs (ERK, JNK and p38 MAPK) and NF-κB have been reported to be the predominant downstream signaling pathways induced by TLR signaling to mediate DC maturation (64). Therefore, we further showed that GL treatment activated ERK, p38 MAPK and NF-κB signaling but had no significant effect on JNK signaling in BMDCs. An inhibitor assay confirmed that GL induced the maturation of BMDCs through the NF-κB, ERK and p38 MAPK signaling pathways. Our present study is anticipated to be beneficial for fully understanding the underlying mechanism of GL in the prevention of *Salmonella* infection in the intestine.

In conclusion, our studies provide substantial evidence confirming that GL regulates gut immunity (lymphocytes and immune factors) and the intestinal flora to attenuate ST infection. This protective mechanism plays an essential role in the prevention of pathogen infection in animals and humans. Given that GL plays pivotal roles in antiviral and antitumor immune responses, we also reveal that GL has anti-inflammatory effects on ST-induced injury in mice and provide a reference for its clinical application in the future.

## Author contributions

All the authors reviewed and approved the final version of the manuscript and agreed to be accountable for the content of the work. WL, XX and YM conceived and designed the experiments. YM, LG, BW and XX performed the experiments. XX, HX and XM analyzed the data. XX, LG, BW, YpW and YW made the figures. XX, LT, LG, BW, RL and ZZ wrote the paper.

### Conflict of interest statement

The authors declare that the research was conducted in the absence of any commercial or financial relationships that could be construed as a potential conflict of interest.

## Supplementary material

Supplemental Figure S1 Dose curve responses of ST and GL in mice. **(A)** C57BL/6 mice were infected with ST at a range of doses 1 × 10^7^, 4 × 10^7^, 1 × 10^8^, 4 × 10^8^, 1 × 10^9^, 4 × 10^9^ CFU. At 36 and 72 h post-infection, body weights were measured respectively. *n* = 6/group. **(B)** Mouse were pretreated with a series of GL gradient concentration during the 21 days before *Salmonella* infection. Body weight changes of the mice were monitored at 36 and 72 h during *Salmonella* infection. *n* = 6/group.

Supplemental Figure S2 (A) Effects of GL on *salmonella typhimurium* (ST) virulence gene expression *in vitro*. ST were resuspended in Luria-Bcrtani (LB) medium and incubated with PBS, GL (100 μg/mL) or GM (25 μg/mL) at 37°C respectively. Total RNA was extracted at 0, 1, 2 and 4 h, and then virulence genes (*ssrB, sipB, hilA, invA*, and *sopD*) expression were measured by real-time PCR. Samples were normalized to the reference gene 16 s rRNA. The data represent three independent experiments. The mean ± SD of the results from three independent experiments is shown. ^*^*P* < 0.05; ^**^*P* < 0.01. **(B)** Schematic of GL protection assay.

Supplemental Figure S3 The relative abundance of the intestinal microbiota was analyzed by sequencing 16S rRNA gene amplicons. Control, PBS treatment; GL, glycyrrhizin pretreatment; ST, *S*. Typhimurium infection; GL plus ST, glycyrrhizin pretreatment and *S*. Typhimurium infection; GM plus ST, gentamicin pretreatment and *S*. Typhimurium infection.

Supplemental Figure S4 Cytotoxicity assay and ACP assay of GL in BMDCs. **(A)** BMDCs were exposed to GL at different concentrations (0, 12.5, 25, 50, 100, 200, 400 and 800 μg/ml) for 48 h. Cell viability was determined by the CCK-8 assay. **(B)** Cell damage was determined by measuring the release of the cytosolic marker LDH. BMDCs were incubated with GL (25, 50, 100 and 200) for 48 h, and LDH release in the supernatant was quantified using the CytoTox96 kit. **(C)** BMDCs were seeded at a density of 5 × 10^5^ cell / well and incubated with PBS (control), GL (25, 50, 100, 200 μg/mL) and LPS (50 ng/mL), for 48 h. ACP activity was detected using the acid phosphatase assay kit. The data are representative of three individual experiments. ^*^*P* < 0.05, ^**^*P* < 0.01 (*t*-test). ns indicates no significance (*P* > 0.05).

Supplemental Figure S5 Flow cytometric analysis of the effect GL on TLR2 expression in BMDCs. BMDCs were treated with PBS (blank control) or GL (200 μg/ml) for 48 h. The cells were stained with antibodies against TLR2, and the fluorescence signals were determined immediately using a FACScan flow cytometer. At least 10,000 events were collected from the cell gate. The data represent three independent experiments. MFI, mean fluorescence intensity.

Supplemental Table S1 Primers used in the experiments.

## References

[1] Pompei R Flore O Marccialis MA Pani A Loddo B. Glycyrrhizic acid inhibits virus growth and inactivates virus particles. Nature (1979) 281:689–90. 10.1038/281689a0 233133

[2] Gong G Xiang L Yuan L Hu L Wu W Cai L. Protective effect of glycyrrhizin, a direct HMGB1 inhibitor, on focal cerebral ischemia/reperfusion-induced inflammation, oxidative stress, and apoptosis in rats. PloS ONE (2014) 9:e89450. 10.1371/journal.pone.0089450 24594628 PMC3942385

[3] Cai Y Zhao B Liang Q Zhang Y Cai J Li G. The selective effect of glycyrrhizin and glycyrrhetinic acid on topoisomerase IIalpha and apoptosis in combination with etoposide on triple negative breast cancer MDA-MB-231 cells. Eur J Pharmacol. (2017) 809:87–97. 10.1016/j.ejphar.2017.05.026 28506909

[4] Zhang X Yang H Yue S He G Qu S Zhang Z. The mTOR inhibition in concurrence with ERK1/2 activation is involved in excessive autophagy induced by glycyrrhizin in hepatocellular carcinoma. Cancer Med. (2017) 6:1941–51. 10.1002/cam4.1127 28675698 PMC5548872

[5] Kimura M Moro T Motegi H Maruyama H Sekine M Okamoto H. *In vivo* glycyrrhizin accelerates liver regeneration and rapidly lowers serum transaminase activities in 70% partially hepatectomized rats. Eur J Pharmacol. (2008) 579:357–64. 10.1016/j.ejphar.2007.10.073 18022618

[6] Zhang YH Kato M Isobe K Hamaguchi M Yokochi T Nakashima I. Dissociated control by glycyrrhizin of proliferation and IL-2 production of murine thymocytes. Cell Immunol. (1995) 162:97–104. 10.1006/cimm.1995.1056 7704916

[7] Ekanayaka SA McClellan SA Barrett RP Kharotia S Hazlett LD. Glycyrrhizin reduces HMGB1 and bacterial load in *Pseudomonas aeruginosa* keratitis. Invest Ophthalmol Vis Sci. (2016) 57:5799–809. 10.1167/iovs.16-20103 27792814 PMC5089214

[8] Steinstraesser L Schubert C Jacobsen F Al-Benna S. Editorial: glycyrrhizin against multi-resistant bacteria? J Leukoc Biol. (2010) 87:7–8. 10.1189/jlb.0809567 20047883

[9] Yoshida T Yoshida S Kobayashi M Herndon DN Suzuki F. Pivotal advance: glycyrrhizin restores the impaired production of beta-defensins in tissues surrounding the burn area and improves the resistance of burn mice to *Pseudomonas aeruginosa* wound infection. J Leukoc Biol. (2010) 87:35–41. 10.1189/jlb.1208760 19843573

[10] Chen JC Ho TY Chang YS Wu SL Li CC Hsiang CY. Identification of Escherichia coli enterotoxin inhibitors from traditional medicinal herbs by *in silico, in vitro*, and *in vivo* analyses. J Ethnopharmacol. (2009) 121:372–8. 10.1016/j.jep.2008.11.011 19063958

[11] Long DR Mead J Hendricks JM Hardy ME Voyich JM. 18beta-Glycyrrhetinic acid inhibits methicillin-resistant Staphylococcus aureus survival and attenuates virulence gene expression. Antimicrobial Agents Chemother. (2013) 57:241–7. 10.1128/AAC.01023-12 23114775 PMC3535912

[12] Ahn SJ Cho EJ Kim HJ Park SN Lim YK Kook JK. The antimicrobial effects of deglycyrrhizinated licorice root extract on Streptococcus mutans UA159 in both planktonic and biofilm cultures. Anaerobe (2012) 18:590–6. 10.1016/j.anaerobe.2012.10.005 23123832

[13] Ohuchi K Kamada Y Levine L Tsurufuji S. Glycyrrhizin inhibits prostaglandin E2 production by activated peritoneal macrophages from rats. Prostaglandins Med. (1981) 7:457–63. 10.1016/0161-4630(81)90033-1 6798590

[14] Takei H Baba Y Hisatsune A Katsuki H Miyata T Yokomizo K Glycyrrhizin inhibits interleukin-8 production and nuclear factor-kappaB activity in lung epithelial cells, but not through glucocorticoid receptors. J Pharmacol Sci. (2008) 106:460–8. 10.1254/jphs.FP0072378 18344608 PMC7129470

[15] Abe M Akbar F Hasebe A Horiike N Onji M. Glycyrrhizin enhances interleukin-10 production by liver dendritic cells in mice with hepatitis. J Gastroenterol. (2003) 38:962–7. 10.1007/s00535-003-1179-7 14614603

[16] Ni YF Kuai JK Lu ZF Yang GD Fu HY Jiang T. Glycyrrhizin treatment is associated with attenuation of lipopolysaccharide-induced acute lung injury by inhibiting cyclooxygenase-2 and inducible nitric oxide synthase expression. J Surg Res. (2011) 165:e29–35. 10.1016/j.jss.2010.10.004 21074783

[17] Jeong HG Kim JY. Induction of inducible nitric oxide synthase expression by 18beta-glycyrrhetinic acid in macrophages. FEBS Lett. (2002) 513:208–12. 10.1016/S0014-5793(02)02311-6 11904152

[18] Yoshida T Tsuda Y Takeuchi D Kobayashi M Pollard RB Suzuki F. Glycyrrhizin inhibits neutrophil-associated generation of alternatively activated macrophages. Cytokine (2006) 33:317–22. 10.1016/j.cyto.2006.03.001 16631375 PMC7128827

[19] Ishiwata S Nakashita K Ozawa Y Niizeki M Nozaki S Tomioka Y. Fas-mediated apoptosis is enhanced by glycyrrhizin without alteration of caspase-3-like activity. Biol Pharm Bull. (1999) 22:1163–6. 10.1248/bpb.22.1163 10598020

[20] Kimura M Watanabe H Abo T. Selective activation of extrathymic T cells in the liver by glycyrrhizin. Biotherapy (1992) 5:167–76. 10.1007/BF02171049 1419465

[21] Nakajima N Utsunomiya T Kobayashi M Herndon DN Pollard RB Suzuki F. *In vitro* induction of anti-type 2 T cells by glycyrrhizin. Burns (1996) 22:612–7. 10.1016/S0305-4179(96)00053-8 8982539

[22] Yoshikawa M Matsui Y Kawamoto H Umemoto N Oku K Koizumi M. Effects of glycyrrhizin on immune-mediated cytotoxicity. J Gastroenterol Hepatol. (1997) 12:243–8. 10.1111/j.1440-1746.1997.tb00416.x 9142643

[23] Bordbar N Karimi MH Amirghofran Z. The effect of glycyrrhizin on maturation and T cell stimulating activity of dendritic cells. Cell Immunol. (2012) 280:44–9. 10.1016/j.cellimm.2012.11.013 23261828

[24] Hua H Liang Z Li W Meng Y Li X Zhang Z. Phenotypic and functional maturation of murine dendritic cells (DCs) induced by purified Glycyrrhizin (GL). Int Immunopharmacol. (2012) 12:518–25. 10.1016/j.intimp.2012.01.006 22293534

[25] Scallan E Hoekstra RM Mahon BE Jones TF Griffin PM. An assessment of the human health impact of seven leading foodborne pathogens in the United States using disability adjusted life years. Epidemiol Infect. (2015) 143:2795–804. 10.1017/S0950268814003185 25633631 PMC9151020

[26] Sharvani R Hemavathi Dayanand DK Shenoy P Sarmah P. Antibiogram of salmonella isolates: time to consider antibiotic salvage. J Clin Diagnost Res. (2016) 10:DC06–08. 10.7860/JCDR/2016/18102.7753 27437211 PMC4948387

[27] Yeom JH Lee B Kim D Lee JK Kim S Lee K. Gold nanoparticle-DNA aptamer conjugate-assisted delivery of antimicrobial peptide effectively eliminates intracellular Salmonella enterica serovar Typhimurium. Biomaterials (2016) 104:43–51. 10.1016/j.biomaterials.2016.07.009 27424215

[28] Xia X Zhang L Wang Y. The antimicrobial peptide cathelicidin-BF could be a potential therapeutic for Salmonella typhimurium infection. Microbiol Res. (2015) 171:45–51. 10.1016/j.micres.2014.12.009 25644952

[29] Kurtz JR Goggins JA McLachlan JB. Salmonella infection: Interplay between the bacteria and host immune system. Immunol Lett. (2017) 190:42–50. 10.1016/j.imlet.2017.07.006 28720334 PMC5918639

[30] Fiorino F Rondini S Micoli F Lanzilao L Alfini R Mancini F. Immunogenicity of a bivalent adjuvanted glycoconjugate vaccine against salmonella typhimurium and salmonella enteritidis. Front Immunol. (2017) 8:168. 10.3389/fimmu.2017.00168 28289411 PMC5326758

[31] Risso GS Carabajal MV Bruno LA Ibanez AE Coria LM Cassataro J. U-Omp19 from brucella abortus is a useful adjuvant for vaccine formulations against salmonella infection in mice. Front Immunol. (2017) 8:171. 10.3389/fimmu.2017.00171 28261222 PMC5313482

[32] Chen X Fang D Li L Chen L Li Q Gong F. Glycyrrhizin ameliorates experimental colitis through attenuating interleukin-17-producing T cell responses via regulating antigen-presenting cells. Immunol Res. (2017) 65:666–80. 10.1007/s12026-017-8894-2 28108937

[33] Taub N Nairz M Hilber D Hess MW Weiss G Huber LA. The late endosomal adaptor p14 is a macrophage host-defense factor against Salmonella infection. J Cell Sci. (2012) 125:2698–708. 10.1242/jcs.100073 22427693

[34] Ji J Hu SL Cui ZW Li WF. Probiotic Bacillus amyloliquefaciens mediate M1 macrophage polarization in mouse bone marrow-derived macrophages. Arch Microbiol. (2013) 195:349–56. 10.1007/s00203-013-0877-7 23455449

[35] Mao Y Wang B Xu X Du W Li W Wang Y. Glycyrrhizic acid promotes m1 macrophage polarization in murine bone marrow-derived macrophages associated with the activation of JNK and NF-kappaB. Mediators Inflamm. (2015) 2015:372931. 10.1155/2015/372931 26664149 PMC4668314

[36] Li Y Wang Y Wu Y Wang B Chen X Xu X. Echinacea pupurea extracts promote murine dendritic cell maturation by activation of JNK, p38 MAPK and NF-kappaB pathways. Dev Comp Immunol. (2017) 73:21–6. 10.1016/j.dci.2017.03.002 28263837

[37] Koh JY Choi DW. Quantitative determination of glutamate mediated cortical neuronal injury in cell culture by lactate dehydrogenase efflux assay. J Neurosci Methods (1987) 20:83–90. 10.1016/0165-0270(87)90041-0 2884353

[38] Traver D Paw BH Poss KD Penberthy WT Lin S Zon LI. Transplantation and *in vivo* imaging of multilineage engraftment in zebrafish bloodless mutants. Nat Immunol. (2003) 4:1238–46. 10.1038/ni1007 14608381

[39] Xu XG Hu JF Ma JX Nie L Shao T Shao J Z. Essential roles of TIM-1 and TIM-4 homologs in adaptive humoral immunity in a zebrafish model. J Immunol. (2016) 196:1686–99. 10.4049/jimmunol.1501736 26792807

[40] Yrlid U Svensson M Johansson C Wick M J. Salmonella infection of bone marrow-derived macrophages and dendritic cells: influence on antigen presentation and initiating an immune response. FEMS Immunol Med Microbiol. (2000) 27:313–20. 10.1111/j.1574-695X.2000.tb01445.x 10727887

[41] Hayman AR Bune AJ Bradley JR Rashbass J Cox TM. Osteoclastic tartrate-resistant acid phosphatase (Acp 5): its localization to dendritic cells and diverse murine tissues. J Histochem Cytochem. (2000) 48:219–28. 10.1177/002215540004800207 10639488

[42] Michelsen KS Aicher A Mohaupt M Hartung T Dimmeler S Kirschning C. The role of toll-like receptors (TLRs) in bacteria-induced maturation of murine dendritic cells (DCS). Peptidoglycan and lipoteichoic acid are inducers of DC maturation and require TLR2. J Biol Chem. (2001) 276:25680–6. 10.1074/jbc.M011615200 11316801

[43] Bansal K Sinha AY Ghorpade DS Togarsimalemath SK Patil SA Bayry J. Src homology 3-interacting domain of Rv1917c of Mycobacterium tuberculosis induces selective maturation of human dendritic cells by regulating PI3K-MAPK-NF-kappaB signaling and drives Th2 immune responses. J Biol Chem. (2010) 285:36511–22. 10.1074/jbc.M110.158055 20837474 PMC2978579

[44] Wang X Zhang H Chen L Shan L Fan G Gao X. Liquorice, a unique “guide drug” of traditional Chinese medicine: a review of its role in drug interactions. J Ethnopharmacol. (2013) 150:781–90. 10.1016/j.jep.2013.09.055 24201019

[45] Maes L Vanden Berghe D Germonprez N Quirijnen L Cos P De Kimpe N. *In vitro* and *in vivo* activities of a triterpenoid saponin extract (PX-6518) from the plant Maesa balansae against visceral leishmania species. Antimicro Agents Chemother. (2004) 48:130–6. 10.1128/AAC.48.1.130-136.2004 14693530 PMC310194

[46] Yadava RN Jharbade J. New antibacterial triterpenoid saponin from Lactuca scariola. Fitoterapia (2008) 79:245–9. 10.1016/j.fitote.2007.11.028 18325685

[47] Coburn B Grassl GA Finlay BB. Salmonella, the host and disease: a brief review. Immunol Cell Biol. (2007) 85:112–8. 10.1038/sj.icb.7100007 17146467

[48] He H Genovese KJ Swaggerty CL Nisbet DJ Kogut MH. A comparative study on invasion, survival, modulation of oxidative burst, and nitric oxide responses of macrophages (HD11), and systemic infection in chickens by prevalent poultry Salmonella serovars. Foodborne Pathog Dis. (2012) 9:1104–10. 10.1089/fpd.2012.1233 23067396

[49] Symonds EL O'Mahony C Lapthorne S O'Mahony D Sharry JM Shanahan F. Bifidobacterium infantis 35624 protects against salmonella-induced reductions in digestive enzyme activity in mice by attenuation of the host inflammatory response. Clin Transl Gastroenterol. (2012) 3:e15. 10.1038/ctg.2012.9 23238232 PMC3367613

[50] Gulig PA Doyle TJ Clare-Salzler MJ Maiese RL Matsui H Systemic infection of mice by wild-type but not Spv- Salmonella typhimurium is enhanced by neutralization of gamma interferon and tumor necrosis factor alpha. Infect Immun. (1997) 65:5191–7. 9393815 10.1128/iai.65.12.5191-5197.1997 PMC175748

[51] Magnusson KE Stendahl O Stjernstrom I Edebo L. Reduction of phagocytosis, surface hydrophobicity and charge of Salmonella typhimurium 395 MR10 by reaction with secretory IgA (SIgA). Immunology (1979) 36:439–47. 374252 PMC1457568

[52] Vitiello M D'Isanto M Finamore E Ciarcia R Kampanaraki A Galdiero M. Role of mitogen-activated protein kinases in the iNOS production and cytokine secretion by Salmonella enterica serovar Typhimurium porins. Cytokine (2008) 41:279–85. 10.1016/j.cyto.2007.11.021 18206384

[53] Forstermann U Sessa WC. Nitric oxide synthases: regulation and function. Eur Heart J. (2012) 33:829–837:837a-837d. 10.1093/eurheartj/ehr304 21890489 PMC3345541

[54] Bogdan C. Nitric oxide and the immune response. Nat Immunol. (2001) 2:907–16. 10.1038/ni1001-907 11577346

[55] Chai D Yue Y Xu W Dong C Xiong S. Mucosal co-immunization with AIM2 enhances protective SIgA response and increases prophylactic efficacy of chitosan-DNA vaccine against coxsackievirus B3-induced myocarditis. Hum Vaccin Immunother. (2014) 10:1284–94. 10.4161/hv.28333 24614684 PMC4896523

[56] Bellussi L Cambi J Passali D. Functional maturation of nasal mucosa: role of secretory immunoglobulin A (SIgA). Multidiscip Respir Med. (2013) 8:46. 10.1186/2049-6958-8-46 23866900 PMC3729437

[57] Stecher B Robbiani R Walker AW Westendorf AM Barthel M Hardt W D. Salmonella enterica serovar typhimurium exploits inflammation to compete with the intestinal microbiota. PLoS Biol. (2007) 5:2177–89. 10.1371/journal.pbio.0050244 17760501 PMC1951780

[58] Hooper LV Gordon JI. Commensal host-bacterial relationships in the gut. Science (2001) 292:1115–8. 10.1126/science.1058709 11352068

[59] Mukherjee S Karmakar S Babu SP. TLR2 and TLR4 mediated host immune responses in major infectious diseases: a review. Braz J Infect Dis. (2016) 20:193–204. 10.1016/j.bjid.2015.10.011 26775799 PMC9427569

[60] Cheng K Gao M Godfroy JI Brown PN Kastelowitz N Yin H. Specific activation of the TLR1-TLR2 heterodimer by small-molecule agonists. Sci Adv. (2015) 1:e1400139. 10.1126/sciadv.1400139 26101787 PMC4474499

[61] Colletti NJ Liu H Gower AC Alekseyev YO Arendt CW Shaw MH. TLR3 Signaling Promotes the Induction of Unique Human BDCA-3 Dendritic Cell Populations. Front. Immunol. (2016) 7:88. 10.3389/fimmu.2016.00088 27014268 PMC4789364

[62] Dalod M Chelbi R Malissen B Lawrence T. Dendritic cell maturation: functional specialization through signaling specificity and transcriptional programming. EMBO J. (2014). 33:1104–16. 10.1002/embj.201488 24737868 PMC4193918

[63] Cuschieri J Billigren J Maier RV. Endotoxin tolerance attenuates LPS-induced TLR4 mobilization to lipid rafts: a condition reversed by PKC activation. J Leukoc Biol. (2006) 80:1289–97. 10.1189/jlb.0106053 16959900

[64] Akira S Uematsu S Takeuchi O. Pathogen recognition and innate immunity. Cell (2006) 124:783–801. 10.1016/j.cell.2006.02.015 16497588

