# Supplementary figures and images for: Glycyrrhizin Attenuates Salmonella enterica Serovar Typhimurium Infection: New Insights Into Its Protective Mechanism
Source: Front Immunol. 2018 Oct 16;9:2321. doi: 10.3389/fimmu.2018.02321 (PMC6232675; doi:10.3389/fimmu.2018.02321)

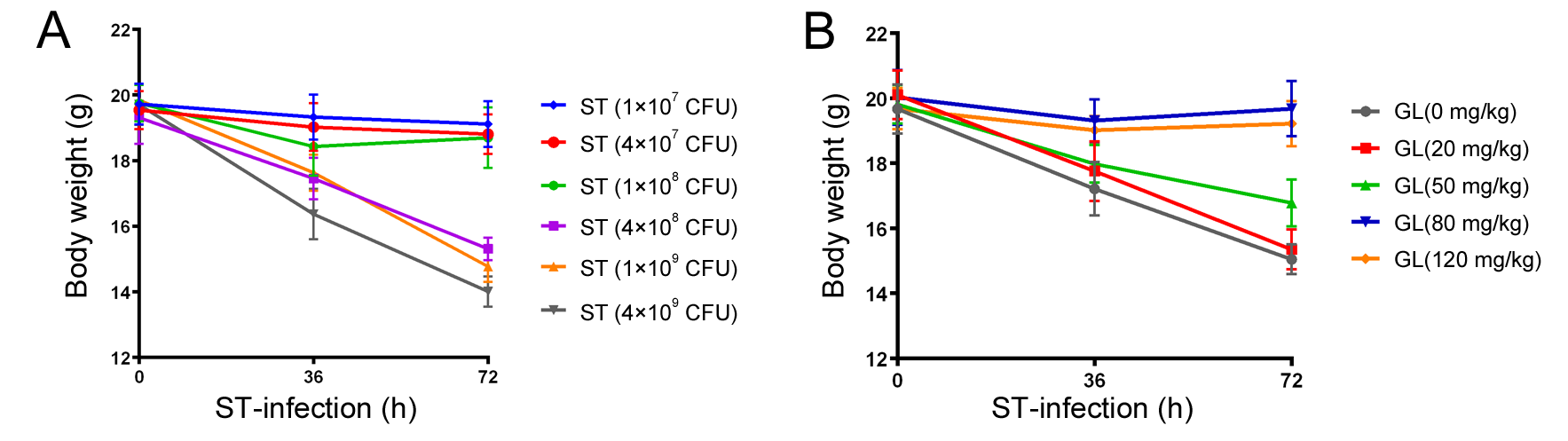

Supplement: Supplemental Figure S1 — Dose curve responses of ST and GL in mice. (A) C57BL/6 mice were infected with ST at a range of doses 1 × 10^7^, 4 × 10^7^, 1 × 10^8^, 4 × 10^8^, 1 × 10^9^, 4 × 10^9^ CFU. At 36 and 72 h post-infection, body weights were measured respectively. n = 6/group. (B) Mouse were pretreated with a series of GL gradient concentration during the 21 days before Salmonella infection. Body weight changes of the mice were monitored at 36 and 72 h during Salmonella infection. n = 6/group. [file Image_1.TIF]

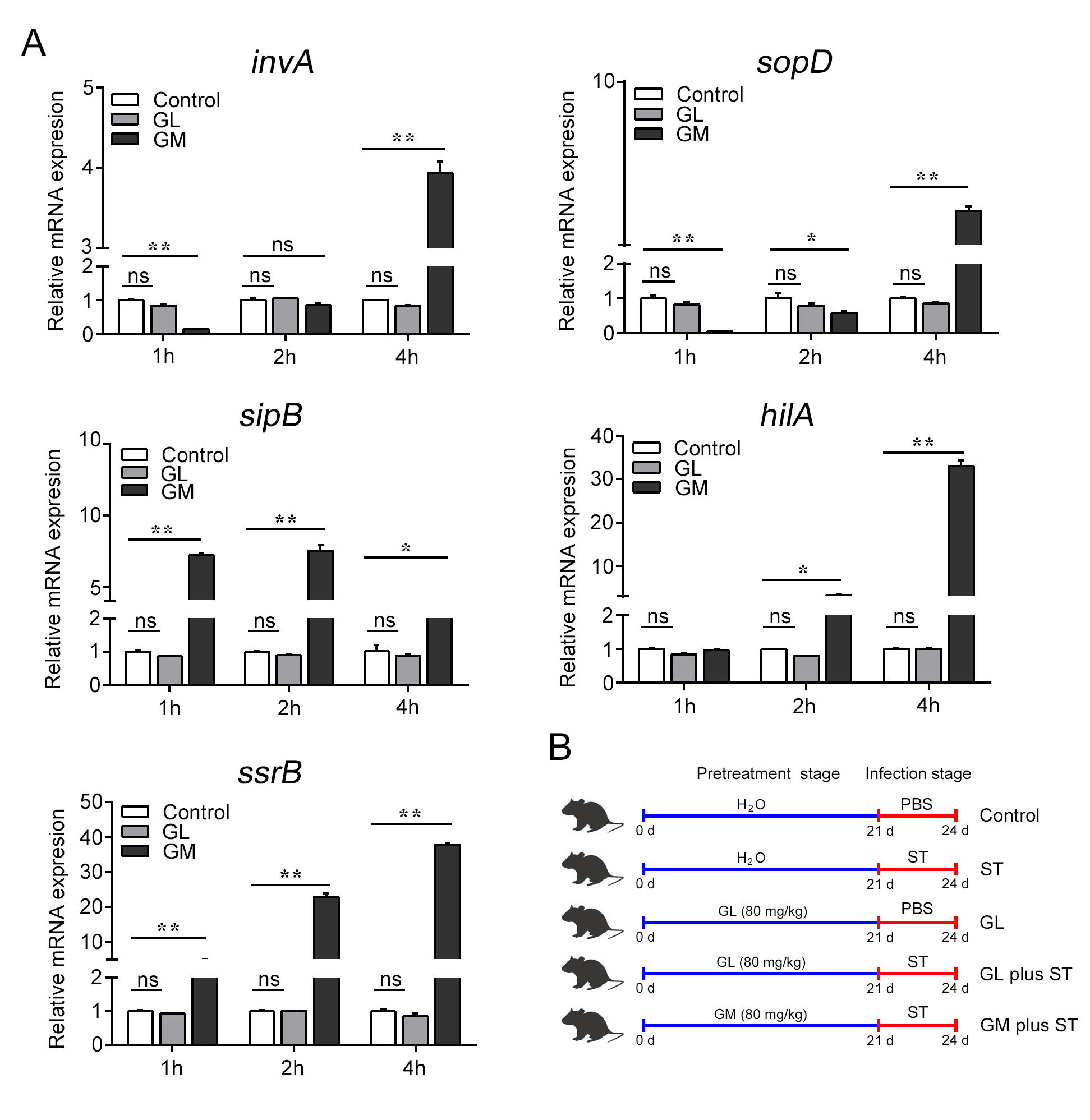

Supplement: Supplemental Figure S2 — (A) Effects of GL on salmonella typhimurium (ST) virulence gene expression in vitro. ST were resuspended in Luria-Bcrtani (LB) medium and incubated with PBS, GL (100 μg/mL) or GM (25 μg/mL) at 37°C respectively. Total RNA was extracted at 0, 1, 2 and 4 h, and then virulence genes (ssrB, sipB, hilA, invA, and sopD) expression were measured by real-time PCR. Samples were normalized to the reference gene 16 s rRNA. The data represent three independent experiments. The mean ± SD of the results from three independent experiments is shown. *P < 0.05; **P < 0.01. (B) Schematic of GL protection assay. [file Image_2.TIF]

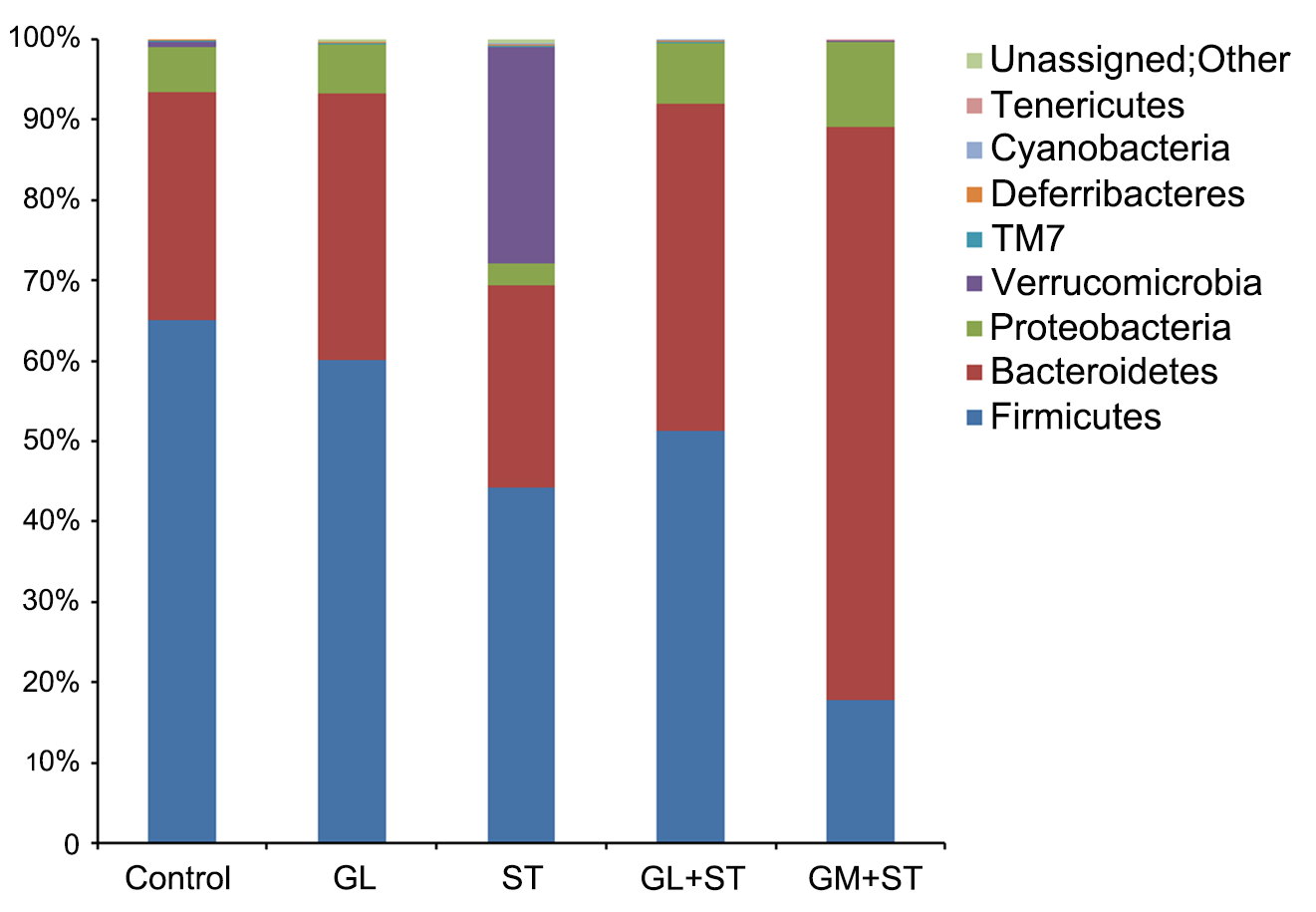

Supplement: Supplemental Figure S3 — The relative abundance of the intestinal microbiota was analyzed by sequencing 16S rRNA gene amplicons. Control, PBS treatment; GL, glycyrrhizin pretreatment; ST, S. Typhimurium infection; GL plus ST, glycyrrhizin pretreatment and S. Typhimurium infection; GM plus ST, gentamicin pretreatment and S. Typhimurium infection. [file Image_3.TIF]

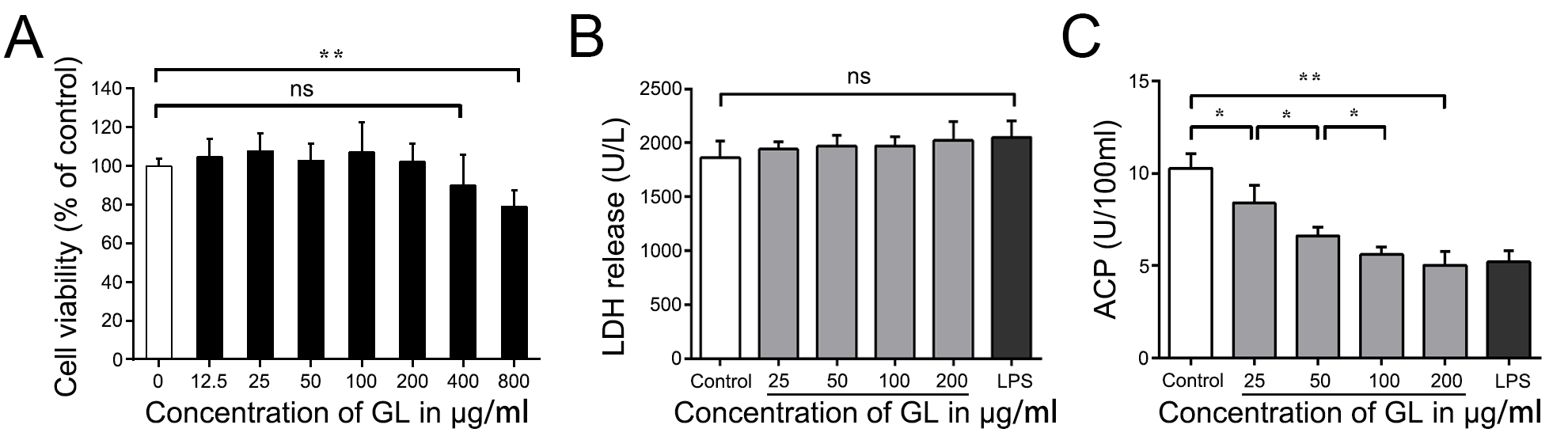

Supplement: Supplemental Figure S4 — Cytotoxicity assay and ACP assay of GL in BMDCs. (A) BMDCs were exposed to GL at different concentrations (0, 12.5, 25, 50, 100, 200, 400 and 800 μg/ml) for 48 h. Cell viability was determined by the CCK-8 assay. (B) Cell damage was determined by measuring the release of the cytosolic marker LDH. BMDCs were incubated with GL (25, 50, 100 and 200) for 48 h, and LDH release in the supernatant was quantified using the CytoTox96 kit. (C) BMDCs were seeded at a density of 5 × 10^5^ cell / well and incubated with PBS (control), GL (25, 50, 100, 200 μg/mL) and LPS (50 ng/mL), for 48 h. ACP activity was detected using the acid phosphatase assay kit. The data are representative of three individual experiments. *P < 0.05, **P < 0.01 (t-test). ns indicates no significance (P > 0.05). [file Image_4.TIF]

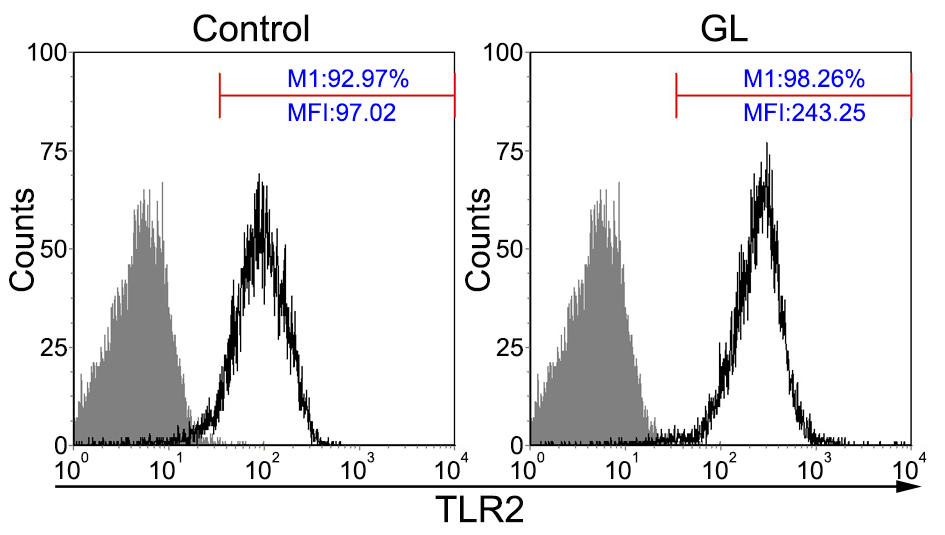

Supplement: Supplemental Figure S5 — Flow cytometric analysis of the effect GL on TLR2 expression in BMDCs. BMDCs were treated with PBS (blank control) or GL (200 μg/ml) for 48 h. The cells were stained with antibodies against TLR2, and the fluorescence signals were determined immediately using a FACScan flow cytometer. At least 10,000 events were collected from the cell gate. The data represent three independent experiments. MFI, mean fluorescence intensity. [file Image_5.TIF]
