# Supplementary material for: Glycyrrhizin Attenuates Salmonella enterica Serovar Typhimurium Infection: New Insights Into Its Protective Mechanism
Source: Front Immunol. 2018 Oct 16;9:2321. doi: 10.3389/fimmu.2018.02321 (PMC6232675; doi:10.3389/fimmu.2018.02321)
Supplement: Supplemental Table S1 — Primers used in the experiments. [file Table_1.docx]

**SUPPLEMENTAL TABLE S1. Primers used in the experiments**

| Gene | Accession Number | Sequence (5’-3’) |
| --- | --- | --- |
| *invA* | M90846 | F: CATTAACCTTGTGGAGCATATTCG  R: CATCCTCAACTTCAGCAGATACC |
| *hilA* | U25352 | F: CGACTCATACATTGGCGATACTT  R: CGGCAGTTCTTCGTAATGGT |
| *sipB* | NC_003197 | F: GTATGGCAGGCGATGATTGA  R: ATAAACACTCTTGGCGGTATCC |
| *sopD* | AF234265 | F: GGACGCTTCTCAGACACAAT  R: CGGGACGCATCATCTCATAA |
| *ssrB* | Z95891 | F: ACGAGCCTGACATACTTATCCT  CGCTAACAGAACTTGCTGACTA |
| *16s rRNA* | NC_003198 | F: CGATGTCTACTTGGAGGTTGTG  R: CTCTGGAAAGTTCTGTGGATGTC |
| *il-4* | NM_021283 | F: AGTTGTCATCCTGCTCTTCTTTC  R: TGGTGTTCTTCGTTGCTGTGA |
| *il-13* | NM_008355 | F: CAACATCACACAAGACCAGACT  R: GAATCCAGGGCTACACAGAAC |
| *tgf-β* | NM_011577 | F: CTCCCGTGGCTTCTAGTGC  R: GCCTTAGTTTGGACAGGATCTG |
| *tlr1* | NM_030682 | F: CAACAGTCAGCCTCAAGCATT  R: CCATAAGCATCTCCTAACACCAG |
| *tlr2* | NM_011905 | F: GCTGGAGGTGTTGGATGTTAG  R: AGGATAGGAGTTCGCAGGAG |
| *tlr3* | NM_126166 | F: CCTATGGATTCTTCTGGTGTCTTC  R: ATCTTCTGAGTTGGTTGTGAGT |
| *tlr4* | NM_021297 | F: GGACTATGTGATGTGACCATTGAT  R: TTATAGATACACCTGCCAGAGACA |
| *tlr5* | NM_016928 | F: GAAGACTGCGATGAAGAGGAAG  R: TACAAGGGTGATGACGAGGAATA |
| *tlr7* | NM_133211 | F: CGGTGATAACAGATACTTGGACTT  R: GATTCTTTAGATTTGGCGGCATAC |
| *tlr9* | NM_031178 | F: GTCAACCTCAGCCACAACAT  R: TGCCACACTTCACACCATTAG |
| *myd88* | NM_010851 | F: CTACAGAGCAAGGAATGTGACT  R: CATATAGTGATGAACCGCAGGAT |
| *trif* | NM_174989 | F: CTCAGCCTCTCATTATTCACCAT  R: CGAACACTCAGTCTTGTCATCA |
